# Predictors of functional disability in people with ischemic stroke: two to three years after the stroke*

**DOI:** 10.1590/1980-220X-REEUSP-2025-0150en

**Published:** 2025-09-19

**Authors:** Brenda Silva Cunha, Mariana de Almeida Moraes, Liane de Assis Campos Medeiros, Ludimila Santos Muniz, Carlos Antônio de Souza Teles Santos, Maria Cecília Bueno Jayme Gallani, Fernanda Carneiro Mussi

**Affiliations:** 1 Universidade Federal da Bahia, Escola de Enfermagem, Salvador, BA, Brazil.; 2 Université Laval, Quebec, QC, Canada.

**Keywords:** Ischemic Stroke, Persons with Disabilities, Thrombolytic Therapy, Rehabilitation, Caregivers, Nursing

## Abstract

**Objective:**

To analyze clinical and sociodemographic factors associated with functional disability in people with ischemic stroke (IS) between 2 and 3 years after the event.

**Method:**

Prospective cohort with 241 participants. Instruments for sociodemographic and clinical characterization, the Rankin-m Scale, and a telephone call protocol were used. Data were analyzed using Pearson’s Chi-square or Fisher’s Exact tests and a robust Poisson model, with a significance level of 5%.

**Results:**

Of the participants, 62.6% had a Rankin of 0 to 2. Multivariate analysis showed that greater neurological deficit (6–13 and ≥14) was 2.35 (95% CI 1.13;4.05) and 4.4 (95% CI 2.09;6.83) times more associated with moderate to severe disability; failure to perform thrombolysis and ischemic stroke recurrence were, respectively, 3.02 (95% CI: 1.32;3.74) and 3.82 times (95% CI: 1.49;3.47) more associated with moderate to severe disability.

**Conclusion:**

The event impacted functional capacity, with greater neurological deficit, non-performance of thrombolysis, and stroke recurrence as the main predictors of disability. Strategies such as expanded thrombolysis, early care, ongoing rehabilitation, and risk factor control can reduce disability.

## INTRODUCTION

Stroke is a leading cause of death, hospitalization, and acquired disability worldwide. Ischemic (IS) and hemorrhagic types are considered clinical emergencies, with IS accounting for approximately 85% of cases^(1)^.

In Brazil, stroke was the second leading cause of death in the years 1990 and 2019. It is estimated that one in four people will have a stroke during their lifetime and that around 17 million cases occur each year in the country, of which approximately one-third results in death^(2)^. Furthermore, hospitalization rates exceeded 185,000 in 2022, generating high costs for the Brazilian Public Health System^(1)^. A study of the *Lancet Neurology Commission* ^(3)^ anticipates that by 2050 deaths could increase by 50%, especially in low- and middle-income countries.

Stroke is the main cause of long-term disability, compromising the social integration of individuals. This impact can lead to early retirement, affecting around 40% of those who have it. It is estimated that approximately 70% of people who have had a stroke are unable to return to their professional activities, and around 50% become dependent on caregivers to carry out daily tasks^(4)^.

This panorama highlights the importance of health promotion, prevention and control of stroke risk factors, as well as the rapid and adequate access to treatment at all levels of care. It is also essential to understand the intrinsic and extrinsic factors influencing the degree of disability, with the aim of minimizing them. Among these factors, clinical and sociodemographic factors stand out^(5)^.

Among clinical factors, the time of treatment is decisive for the prognosis. Therapies are time-dependent, and thrombolysis, when administered within 4.5 hours of symptom onset, reduces mortality and the level of disability^(6)^. Moreover, the initial severity of the stroke, measured using the *National Institute of Health Stroke Scale* (NIHSS), is associated with the degree of functional disability, with higher scores indicating greater neurological impairment^(7)^. Other factors, such as the presence of diabetes, high blood pressure, excessive alcohol consumption, a sedentary lifestyle, atrial fibrillation and other heart and lung diseases, confer an increased risk of functional disability^(8)^. There is evidence that COVID-19 can interfere with the immune response, causing systemic inflammation, which can aggravate brain injury after an IS and, consequently, increase the degree of disability^(9)^.

Additionally, the time since the onset of the ischemic stroke may influence the functional prognosis, with recovery being more significant in the first weeks and between three and six months after the event^(10)^. Early initiation of rehabilitation helps reduce brain damage and improve functionality. Furthermore, hospitalization in Stroke Units, which have adequate structure and specialized staff, is associated with lower mortality and functional dependence, providing better outcomes upon hospital discharge^(11)^. The importance of secondary prophylaxis, which includes the use of antiplatelet therapy to prevent clot formation and reduce the risk of recurrence^(12)^ should also be emphasized. In cases of atrial fibrillation, anticoagulants are used, while statins are indicated to control dyslipidemia, prevent new events, and improve long-term outcomes^(13)^. Controlling high blood pressure is also essential, and is achieved through specific drug therapy^(14)^.

Among the sociodemographic factors related to functional disability due to IS, increasing age, female gender, and living with a partner stand out^(15,16,17)^. Some indicators of social inequality such as self-declared black race/color, low education, and income^(17)^ are also pointed out as predictors.

Despite knowledge of the aforementioned factors associated with functional disability, the analysis of these predictors is still incipient, especially in Brazil, including different times of follow up. Furthermore, mortality and functional disability due to ischemic stroke, especially in developing countries such as Brazil and in regions of the country such as the Northeast, with unfavorable socioeconomic indicators, lack short- and long- term records.

Knowing the degree of disability and its predictors over time after ischemic stroke provides relevant information to improve healthcare management, direct treatment, nursing care practices, and public policies aimed at people with IS. In addition, it can guide the healthcare team in health promotion, prevention, control, and rehabilitation actions.

Based on the above, the present study aimed to analyze the clinical and sociodemographic factors associated with functional disability in people with IS between two and three years after the ictus.

## METHOD

### Study Design

This is a prospective and analytical cohort study^(18)^.

### Study Local

The research was carried out in a public, high complexity, tertiary General Hospital, of an assistance nature, with 640 beds, in the state of Bahia.

### Sample

This study is part of the Matrix Project “Factors associated with disability and mortality due to Ischemic Stroke and times of access to treatment”.

During the baseline period of the study, 320 people with ischemic stroke were admitted to the study site, to whom the inclusion criteria were applied (diagnosis of acute IS clinically confirmed by the physician and recorded in the medical record with compatible imaging and age ≥ 18 years) and the exclusion criteria (signs and symptoms that made verbal communication impossible for the participant in the absence of a companion to answer the research questions and time since stroke greater than 10 days, due to the possibility of recall bias). Of the 320 participants recruited, 12 met the exclusion criteria, making a sample of 308 followed through daily visits during hospitalization and, subsequently, by telephone call up to 90 days after the stroke. Of the 308 participants, 38 died in the hospital and 20 after discharge. Nine were lost to follow-up, totaling 242 survivors within 90 days of the event, who were recontacted by phone between two and three years after the stroke, from March 2021 to November 2023. Among them, 33 died, 54 were lost to follow-up, and 155 constituted the sample of the present study. For this study, death was an exclusion criterion.

### Data Collection Instruments

An instrument was used for sociodemographic and clinical characterization and to identify the arrival time at a neurology referral hospital (ATRH) in hours.

A telephone call protocol was also used, containing items to record the participant’s identification, the score obtained by applying the modified Rankin Scale (mRS), the occurrence of death, new stroke and Covid-19, self-reported use of medication for secondary prophylaxis, and follow-up consultations with a physiotherapist, doctor, nurse, or other health professionals.

The mRS is widely used in clinical practice and research to assess the degree of functional disability related to activities of daily living, with an emphasis on motor impairment, in the face of stroke. The version used was translated and culturally adapted for Brazil and validated for administration by telephone^(19)^. The degree of disability is classified on a scale of 0 to 6 points, namely: 0 = asymptomatic, no disability; 1 = no significant disability; 2 = mild disability; 3 = moderate disability; 4 = moderately severe disability; 5 = severe disability; and 6 = death^(19)^.

### Data Collection Procedures

Sociodemographic and clinical data were obtained through interviews, except for the admission NIHSS, the presence of atrial fibrillation (AF), diabetes mellitus (DM), arterial hypertension (AH), dyslipidemia and thrombolysis, which were collected from the medical records. In situations where the eligible participant did not have the clinical, cognitive, and/or emotional conditions to interact with the researcher, the approach was made to his/her companion. The date and time of onset of symptoms or whether there was Wake Up Stroke were asked to the affected person and/or companion at the time of the interview. The date and time of arrival at the study site were retrieved from the medical record.

The telephone calls between two and three years after the stroke were made using smartphones, by two undergraduate students and one master’s student, who were properly trained by a project researcher that made the follow-up calls within 90 days of the stroke. In this phase, the responses to the instruments used were given, preferably, by the participant him/herself and, if this was not possible due to impaired communication and understanding conditions, by the person who accompanied them in their daily activities. Three call attempts were made on different days and times of the week. If one of the contactees was unsuccessful, the instrument was given to another one so that two more attempts could be made to avoid as many losses of study participants as possible. In case of unsuccessful telephone contact after five attempts, follow-up was considered lost.

Every participant had a printed telephone call protocol, which was in the hands of the interviewers at the time of the call, which also contained their number corresponding to the database. After recording the data collected in a physical protocol, all were transferred to the database in SPSS version 22.0 and the physical protocols were properly stored for possible consultations.

### Data Analysis

The independent (sociodemographic and clinical) variables and the dependent variable (functional disability) were analyzed in absolute and relative frequencies. For bivariate and multivariate analyses, the functional disability variable was dichotomized into: 0 to 2 (asymptomatic to mild disability) and 3 to 5 (moderate to severe disability). To verify the association between functional disability and the variables of interest, Pearson’s Chi-square test or Fisher’s exact test was used. Next, the variables whose associations showed a p-value ≥ 20 were included in the multivariate analysis. Considering the high proportion of severe to moderate disability in the sample, the overestimation of *Odds Ratio* was corrected, through the calculation of the RP estimate and its respective 95% confidence interval, using the robust Poisson regression model.

Modeling was performed using the backward procedure. To choose the best model, the Akaike Information Criterion (AIC) was used and the one with the lowest value was chosen. At this stage, the statistical significance adopted was 5%. Data were processed and analyzed using the software *Statistical Package for the Social Sciences* (SPSS), version 22, and STATA version 12.

### Ethical Aspects

The study is part of the Matrix Project and was approved by the institution’s Ethics Committee, opinion numbers 3,159,694 and 6,666,572. Participants were assured of confidentiality of personal identity, privacy, and the right to withdraw from the research at any stage, receiving clarification on the study objectives and procedures and of any questions regarding the content of the Free and Informed Consent Form.

## RESULTS

In the sample of 155 participants, it was observed that 62.6% were asymptomatic or had mild disability, while 37.4% had moderate to severe disability. Regarding sociodemographic variables, the predomination was of women (50.3%), age group from 60 to 79 years (55.5%), marital status married/with partner (52.3%), unfinished/finished primary education (49.0%), inactive employment status (73.5%), monthly family income of up to one minimum wage (45.0%), and self-declared brown race-color (55.2%). A higher percentage of women with moderate to severe disability (p = 0.003) and a higher percentage of participants without work activity presenting greater disability (p = 0.013) were observed. The other sociodemographic variables were independent of the degree of disability (Table 1).

**Table 1:** Association between sociodemographic variables and degree of functional disability between two and three years after stroke – Salvador, Bahia, Brazil, 2023.

Sociodemographic variables	Total n (%)	Degree of functional disability		p-value
		Rankin 0–2 n (%)	Rankin 3–5 n (%)	
Sex				
Female	78 (50.3)	40 (51.3)	38 (48.7)	0.003*
Male	77 (49.7)	57 (74.0)	20 (26.0)	
Age				
Up to 59 years old	53 (34.2)	32 (60.4)	21 (39.6)	0.429**
60 to 79 years old	86 (55.5)	57 (63.3)	29 (33.7)	
≥ 80 years old	16 (10.3)	8 (50.0)	8 (50.0)	
Marital status				
Married/with partner	81 (52.3)	52 (64.2)	29 (32.8)	0.663**
Single/widowed/separated/Divorced	74 (47.7)	45 (60.8)	29 (39.2)	
Level of education				
Finished or unfinished higher education	10 (6.5)	6 (60.0)	4 (40.0)	0.988**
Finished or unfinished high school education	48 (31.0)	31 (64.6)	17 (35.4)	
Finished or unfinished elementary education	76 (49.0)	47 (61.8)	29 (38.2)	
Illiterate/signs own name	21 (13.5)	13 (61.9)	8 (38.1)	
Employment situation				
With activity	68 (43.9)	50 (73.5)	18 (26.5)	0.013*
Without activity	87 (56.1)	47 (54.0)	40 (46.0)	
Family income in minimum wages (n = 149)				
>3	19 (12.8)	14 (73.7)	5 (26.3)	0.517*
> 1 to 3	63 (42.2)	38 (60.3)	25 (39.7)	
Uo to one	67 (45.0)	40 (59.7)	27 (40.3)	
Race/color (n = 154)				
White	22 (14.3)	14 (63.6)	8 (36.4)	0.570**
Black	47 (30.5)	32 (68.1)	15 (31.9)	
Brown	85 (55.2)	50 (58.8)	35 (41.2)	

* P-value obtained by Pearson’s Chi-square

**P-value obtained by Fisher’s Exact test.

Regarding clinical variables, 45.4% presented NIHSS characteristic of moderate neurological deficit and 35.4% mild deficit, 59.4% arrived at the study site within 4.5 h of the onset of symptoms or wake-up stroke, 72.9% did not undergo thrombolysis, 76.1% had hypertension, 73.4% had no previous stroke (73.4%) nor AF (92.6%). Furthermore, the majority did not smoke (60.6%), did not have diabetes (76.6%), dyslipidemia (69.1%), myocardial infarction (91.0%), COVID-19 (91.6%), nor new stroke between two and three years after the ictus (92.3%) (Table 2). It was observed that higher NIHSS scores (p = 0.000), previous stroke (p = 0.036), and the occurrence of new stroke between two and three years after the last event (p = 0.001) were associated with moderate to severe disability. The variables DM, dyslipidemia, AMI, smoking, AF, Covid-19, ATRH, and submission to thrombolysis were independent of disability.

**Table 2:** Association between clinical variables and functional disability between two and three years after the stroke – Salvador, Bahia, Brazil, 2023.

Clinical variables	Total n (%)	Degree of functional disability		p-value*
		Rankin 0–2 n (%)	Rankin 3–5 n (%)	
NIHSS (n = 130)				
≤5	46 (35.4)	36 (78.3)	10 (21.7)	0.000
6 to 13	59 (45.4)	37 (62.7)	22 (37.3)	
≥14	25 (19.2)	7 (28.0)	18 (72.0)	
Admission to Stroke Unit				
Yes	120 (77.4)	75 (62.5)	45(37.5)	0.969
No	35 (22.6)	22 (62.9)	13 (37.1)	
ATRH				
≤ 4.5h	92 (59.4)	59 (64.1)	33 (35.9)	0.630
> 4.5h	63 (40.6)	38 (60.3)	25 (39.7)	
Thrombolysis				
Yes	42 (27.1)	31 (73.8)	11 (26.2)	0.078
No	113 (72.9)	66 (58.4)	47 (41.6)	
Previous stroke (n = 154)				
No	113 (73.4)	76 (67.3)	37 (32.7)	0.036
Yes	41 (26.6)	20 (48.8)	21 (51.2)	
Atrial fibrillation (n = 148)				
No	137 (92.6)	86 (62.8)	51 (37.2)	0.588
Yes	11 (7.4)	6 (54.5)	5 (45.5)	
Diabetes Mellitus (n = 154)				
No	118 (76.6)	77 (65.3)	41 (34.7)	0.291
Yes	36 (23.4)	20 (55.6)	16 (44.4)	
High blood pressure				
No	37 (23.9)	28 (75.7)	9 (24.3)	0.059
Yes	118 (76.1)	69 (58.5)	49 (41.5)	
Dyslipidemia				
No	107 (69.1)	70 (65.4)	37 (34.6)	0.275
Yes	48 (30.9)	27 (56.3)	21 (43.8)	
Myocardial Infarction				
No	141 (91.0)	89 (63.1)	52 (36.9)	0.659
Yes	14 (9.0)	8 (57.1)	6 (42.9)	
Smoking				
No	94 (60.6)	61 (64.9)	33 (35.1)	0.491**
Yes	16 (10.4)	11 (68.8)	5 (31.3)	
Former smoker	45 (29.0)	25 (55.6)	20 (44.4)	
COVID-19 (n = 154)				
No	141 (91.6)	89 (63.1)	52 (36.9)	0.595
Yes	13 (8.4)	7 (53.8)	6 (46.2)	
New stroke				
No	143 (92.3)	95 (66.4)	48 (33.6)	0.001*
Yes	12 (7.7)	2 (16.7)	10 (83.3)	
Secondary prophylaxis (n = 132)				
Yes	83 (53.6)	54 (65.1)	29 (34.9)	0.783*
No	49 (31.6)	29 (59.2)	20 (40.8)	
Don&apos;t know	23 (14.8)	14 (60.9)	9 (39.1)	

* P-value obtained by Pearson’s Chi-square

**P-value obtained by Fisher’s Exact test.

In the bivariate analyses, the variables presenting a statistically significant difference of up to 20% (sex, work activity, NIHSS, thrombolysis, previous stroke, SAH and new stroke) were tested as predictors for the degree of disability in the multivariate analysis.

The final model showed that participants with NIHSS scores of 6 to 13 and ≥14 points presented, respectively, 2.4 times (PR 2.35, 95% CI 13–4.05) and 4.4 times more moderate to severe disability (PR 4.40, 95% CI: 2.09–6.83) compared to those with scores from 0 to 5. Those who did not undergo thrombolysis and had a new stroke between two and three years after the ictus presented, respectively, 3.0 times (PR = 3.02, 95% CI 1.32–3.74) and 3.8 times (PR 3.82, 95% CI 1.49–3.47) more moderate to severe disability compared to those who received thrombolytic therapy and suffered a new event (Table 3).

**Table 3:** Multivariate analysis with variables associated with functional disability between two and three years after the stroke – Salvador, Bahia, Brazil, 2023.

Variables	PR (CI 95%)
NIHSS	
≤5	1.00
6 to 13	2.35 (1.13–4.05)
≥14	4.40 (2.09–6.83)
Thrombolysis	
Yes	1.0
No	3.02 (1.32–3.74)
New stroke	
No	1.0
Yes	3.82 (1.49–3.47)
Akaike Information Criterion = 185.3753	

The results showed that, between two and three years after the ischemic stroke, most participants were asymptomatic or had mild dysfunction and that clinical factors such as greater initial neurological deficit, absence of thrombolysis, and ischemic stroke recurrence were the main contributors to moderate to severe functional disability.

## DISCUSSION

The study showed the impact of IS on the lives of those affected, as more than a third presented moderate to severe disability between two and three years after the stroke. In general, people with a greater degree of disability have greater difficulty performing daily tasks independently and require assistance from caregivers or some type of equipment to help them perform these activities, in addition to needing the support of a multidisciplinary team^(20)^.

A study conducted in Shanghai, China (n = 305), assessed functional disability after ischemic stroke using the modified Rankin Scale (mRS), and observed a higher proportion of asymptomatic individuals or individuals with mild disability between one month and two years after the event (83.2%). Only 16.8% had moderate to severe disability, a percentage lower than that identified in the present study. In the Chinese study, lower degrees of functional disability were associated with cognitive impairment, the presence of depressive symptoms, and a previous history of stroke—a fact that, although not statistically significant in our sample, may be related to worse outcomes^(21)^.

The sample was mostly made up of elderly people and long-lived elderly people who have a greater chance of IS when living with diseases that affect the cardiovascular system. It is estimated that the risk of IS doubles every 10 years, starting at age 55^(22)^. Our results corroborate another study^(21)^, showing that 77.1% had a stroke between 61 and 80 years of age. Despite the independence between age and disability, the importance of prevention and control of risk factors for the event at all stages of life is highlighted.

Ischemic stroke affected similar proportions between the sexes, with a predominance of cases in women, a finding that corroborates data from a national study^(15)^. This greater incidence in females may be related to higher lipid levels, menopause, and the impact of aging on vascular health. In the present study, women presented, in greater proportion, moderate to severe disability, and are more likely to progress to an unfavorable prognosis, with an increased risk of death^(23)^.

Most study participants lived with a partner. Considering the high percentage of individuals with moderate to severe disability, there is a significant dependence on social support and assistance with self-care. Married people or those in a common-law marriage tend to receive more emotional and practical support from their spouses during recovery from ischemic stroke, which can favor adherence to medical recommendations and the adoption of a healthy lifestyle, contributing to a more favorable prognosis^(16)^. However, in the present study, the variables marital status and degree of disability were shown to be independent.

It is worth highlighting that the nurse can identify the spouses of IS victims who can provide support and be active in planning, supervising and implementing home care, as well as in providing resources, motivating and monitoring the rehabilitation process. In a situation of social inequality, as the majority of the sample found themselves, the challenge of post-stroke care is even greater. The sample was predominantly made up of black and brown people, with low education, low income and no work activity. Low education levels can result in low income, less knowledge about risk factors and, consequently, less prevention and control of the disease^(17)^. White people, in general, have greater access to rehabilitation after stroke compared to black and brown people^(17)^. In our study, despite the lack of statistical significance between income and disability, a higher percentage of participants with lower economic power was found with severe disability. Therefore, it is necessary to ensure, in the rehabilitation process, continuous access to public services for groups in situations of socioeconomic disadvantage, to ensure the effectiveness of their recovery and social inclusion.

Participants without work activity presented moderate to severe disability in greater proportion. A study of Japanese people showed an increased risk of morbidity and mortality from stroke among those who spent at least one period without working or lost their jobs compared to those who were active at work^(24)^. It is possible to hypothesize that the active work situation provides physical and cognitive stimulation with recovery of skills and improvement of the emotional status.

Regarding clinical variables, a high percentage of participants had high blood pressure, an independent risk factor for stroke and its recurrence. These ones presented a greater proportion of moderate to severe disability, with a statistical borderline difference in bivariate analysis. Arterial hypertension is associated with a worse functional prognosis, as it increases the risk of cerebral edema and intracerebral hemorrhage. Hypertensive individuals affected by ischemic stroke tend to present greater severity of the initial neurological deficit, assessed by the NIHSS scale, in addition to greater extent of brain injury and higher rates of functional disability compared to normotensive individuals^(25)^.

A smaller proportion of participants had atrial fibrillation, while diabetes mellitus affected approximately a quarter of the sample and dyslipidemia, approximately a third. These clinical conditions are associated with a higher risk of morbidity and mortality from IS, as they potentiate micro and macrovascular damage^(26)^, and require continuous clinical management. However, in the present study, such variables were shown to be independent of the degree of functional disability. The importance of accurate diagnosis of atrial fibrillation after IS is emphasized, as it allows the implementation of specific secondary prevention strategies, such as the use of anticoagulants, which contribute to reducing the risk of recurrence^(27)^.

During the follow-up period, some participants were diagnosed with COVID-19; however, no association was observed between the infection and the degree of functional disability. However, the literature indicates that individuals who contract COVID-19 after a stroke are more likely to have the event recur, more severe forms of the disease, and cardiovascular complications^(28)^.

Approximately one-third of the sample had a history of stroke, which was associated with a higher degree of disability only in the bivariate analysis. On the other hand, recurrence of IS during the follow-up period was one of the three variables significantly associated with moderate to severe disability in the multivariate analysis, increasing the chance of functional impairment by 3.82 times. Corroborating these findings, Yang and contributors^(29)^ demonstrated, through multiple logistic regression, that stroke recurrence is a predictor of a greater degree of disability. Ischemic accounts for acute disabilities that tend to stabilize after a period of three to six months. However, stabilization is compromised if new events occur.

A previous history of stroke and its recurrence, observed in our study, can lead to worsening gait-related deficits and impaired motor function due to new brain lesions^(29)^. The recurrence of ischemic stroke within two and three years after the ictus highlighted the difficulties in preventing and controlling the disease and the importance of continued monitoring of affected individuals with a multidisciplinary team, assessment, and support for adherence to therapy. A prospective cohort study with 764 patients observed that 18.0% had a new ischemic stroke in the first three years after discharge, corroborating our results, with lack of adherence to secondary prophylaxis, after the first event, being a risk factor for recurrence^(12)^.

In the present investigation, moderate neurological deficit assessed by NIHSS was more frequent. On the scale, as the score increases, it expresses greater neurological impairment and disability, which translates into unfavorable results for quality of life^(16)^. In the bivariate analysis, moderate to severe disability increased proportionally with the severity of the event. Multivariate analysis made the relationship established between the severity of the neurological deficit and the degree of disability more evident, as people with moderate and severe neurological deficit had, respectively, 2.35 and 4.40 times more moderate to severe disability compared to those with mild disability. A five-year cohort evaluated people with and without functional disability after an IS, showing that 45% of the sample (n = 893) remained with functional disability after this period and that a higher NIHSS was significantly associated with a greater degree of disability, corroborating our findings^(29)^. Another study, carried out with 101 people who suffered an IS, evaluated at one, three and six months after the event, observed that after six months of the ictus, 50% were unable to recover their functions and had higher scores on the NIHSS^(16)^.

Most participants arrived at the study site within the therapeutic window; however, less than 30% underwent thrombolysis, which contributed to a higher degree of disability, as shown by multivariate analysis. Participants not undergoing thrombolysis had 3.0 times more moderate to severe disability compared to those who received thrombolytic treatment, demonstrating its benefits two and three years after the event. Furthermore, a significant percentage arrived outside the therapeutic window, failing to enjoy its benefits. Currently, IS guidelines limit initiation of intravenous thrombolytic therapy to 4.5 hours after symptom onset or wake-up stroke. The use of alteplase therapy shows that those who benefited had minimal or no neurological deficits^(1)^. Other authors have reinforced the positive effects of thrombolysis, helping to reduce disability, increasing the likelihood of a faster return to work, and promoting an improvement in quality of life^(30)^.

The importance of nurses’ role in supporting patients and families from the beginning of the rehabilitation process is emphasized, especially during the transition phase from hospital to home. In this context, it is up to the professional to identify potential caregivers and train them to provide adequate support to the patient. This process requires adaptation on the part of both the patient and the family, especially in cases of greater degrees of disability. The guidelines shared by the nurse in the hospital environment are essential not only for home care, but also to ensure continuity of monitoring in primary care, promoting the connection with the health unit^(31)^.

The results of the present study indicated that greater neurological deficit, absence of thrombolysis, and recurrence of ischemic stroke were the main factors associated with moderate to severe disability. These findings highlight the importance of public policies aimed at caring for people affected by ischemic stroke, as well as effective rehabilitation strategies in all phases of treatment, with the aim of minimizing the degree of functional disability. The need for studies focused on the longitudinal monitoring of these individuals by multidisciplinary teams in the public health system is reinforced, ensuring universal access to quality rehabilitation and effective secondary prevention actions.

The results reinforce the importance of adhering to consolidated prevention strategies, such as the HEARTS initiative, which represents a fundamental link in the prevention of cerebrovascular diseases. It is an acronym that encompasses six essential pillars: healthy habits; evidence-based clinical protocols; access to medicines, technologies, and supplies; cardiovascular risk management; multidisciplinary teamwork; and monitoring through standardized indicators. The effective implementation of this strategy can significantly contribute to better results in the prevention and control of ischemic stroke.

The findings of this research also highlight the need to expand the coverage of the public health system regarding the use of thrombolytic therapy, which is effective in reducing more severe neurological dysfunctions. At the same time, it is critical that the community and affected individuals are guided to recognize the signs and symptoms of ischemic stroke early, to seek care in neurology reference units within the therapeutic window. In this study, approximately 40% of participants arrived at the hospital service outside the therapeutic window. Here lies a fundamental space for nurses to work through health education activities. Moreover, a country with fewer social disparities would be able to ensure better living conditions, minimizing illness and the resulting suffering.

This study has some limitations that should be considered when interpreting the results. There was a loss of participants during follow-up, which may compromise the representativeness of the final sample. Additionally, data collection through telephone interviews and, in some cases, where data were provided by caregivers and family members, may have generated some information bias. Another bias to be considered refers to the selection of participants at baseline, since some patients in more severe conditions or with cognitive deficits without companions available at the time of inclusion were excluded, which may limit the generalization of the findings to the population with greater functional impairment.

## CONCLUSION

The study showed the impact of IS on the lives of those affected, as more than a third presented moderate to severe disability between two and three years after the stroke. The variables associated with moderate to severe disability, in the multivariate analysis, were greater severity of neurological deficit, failure to undergo thrombolysis, and recurrence of IS. The study highlights the need for improvements in the healthcare network, ensuring increased provision of thrombolysis, access to specialized treatment units, and early and ongoing rehabilitation with a multidisciplinary team. It emphasizes that effective policies and interventions aimed at promoting health, preventing and controlling risk factors for ischemic stroke are essential, as well as encouraging early referral of victims to a neurology reference hospital to minimize the negative repercussions of ischemic stroke on personal and family life and on society in general.

## Data Availability

The dataset supporting the results of this study is not publicly available.
