# Supplementary material for: Predictors of functional disability in people with ischemic stroke: two to three years after the stroke
Source: Rev Esc Enferm USP. 2025 Sep 19;59:e20250150. doi: 10.1590/1980-220X-REEUSP-2025-0150en (PMC12448757; doi:10.1590/1980-220X-REEUSP-2025-0150en)
Supplement: Translation

# Translation: Preditores de incapacidade funcional em pessoas com acidente vascular cerebral isquêmico: dois a três anos do ictus*

**DOI:** 10.1590/1980-220X-REEUSP-2025-0150pt

**Authors:** Brenda Silva Cunha, Mariana de Almeida Moraes, Liane de Assis Campos Medeiros, Ludimila Santos Muniz, Carlos Antônio de Souza Teles Santos, Maria Cecília Bueno Jayme Gallani, Fernanda Carneiro Mussi

**Affiliations:** 1 Universidade Federal da Bahia, Escola de Enfermagem, Salvador, BA, Brasil.; 2 Université Laval, Quebec, QC, Canada.

**Keywords:** AVC Isquêmico, Pessoas com Deficiência, Terapia trombolítica, Reabilitação, Cuidadores, Enfermagem

## Abstract

**Objetivo:**

Analisar fatores clínicos e sociodemográficos associados à incapacidade funcional em pessoas com acidente vascular cerebral isquêmico (AVCI) entre 2 e 3 anos do evento.

**Método:**

Coorte prospectiva com 241 participantes. Foram utilizados instrumentos para caracterização sociodemográfica e clínica, a Escala de Rankin-m e um protocolo de ligação telefônica. Os dados foram analisados com testes Qui-quadrado de Pearson ou Exato de Fisher e Modelo de Poisson robusto, com significância de 5%.

**Resultados:**

Dos participantes, 62,6% apresentaram Rankin de 0 a 2. A análise multivariada mostrou que maior déficit neurológico (6–13 e ≥14) foi 2,35 (IC 95% 1,13;4,05) e 4,4 (IC 95% 2,09; 6,83) vezes mais associado a incapacidade moderada a grave; não realização de trombólise e recorrência do AVCi foram, respectivamente, 3,02 (IC 95%: 1,32;3,74) e 3,82 vezes (IC 95%: 1,49;3,47) mais associados a incapacidade moderada a grave.

**Conclusão:**

O evento impactou a capacidade funcional, com maior déficit neurológico, não realização de trombólise e recorrência do AVC como principais preditores de incapacidade. Estratégias como ampliação da trombólise, atendimento precoce, reabilitação contínua e controle de fatores de risco podem reduzir as incapacidades.

## INTRODUÇÃO

O acidente vascular cerebral (AVC) é uma das principais causas de morte, hospitalização e incapacidade adquirida em todo o mundo. Os tipos isquêmico (AVCi) e hemorrágico são considerados emergências clínicas, sendo o AVCi responsável por cerca de 85% dos casos^(1)^.

No Brasil, o AVC foi a segunda principal causa de óbito nos anos de 1990 e 2019. Estima-se que uma em cada quatro pessoas sofrerá um AVC ao longo da vida e que cerca de 17 milhões de casos ocorram por ano no país, dos quais aproximadamente um terço evolui para óbito^(2)^. Além disso, as taxas de internação ultrapassaram 185 mil em 2022, gerando gastos elevados para o Sistema Único de Saúde^(1)^. Estudo da *Lancet Neurology Commission* ^(3)^ prevê que até 2050 as mortes podem aumentar em 50%, principalmente nos países de baixa e média renda.

O AVC é a principal causa de incapacidade em longo prazo, comprometendo a integração social dos indivíduos. Esse impacto pode levar à aposentadoria precoce, afetando cerca de 40% dos acometidos. Estima-se que aproximadamente 70% das pessoas que sofreram um AVC não conseguem retomar suas atividades profissionais, e cerca de 50% passam a depender de cuidadores para realizar tarefas cotidianas^(4)^.

Esse panorama evidencia a importância da promoção da saúde, da prevenção e do controle dos fatores de risco para o AVC, além do acesso rápido e adequado ao tratamento em todos os níveis de atenção. Também é fundamental compreender os fatores intrínsecos e extrínsecos que influenciam o grau de incapacidade, com o objetivo de minimizá-los. Entre esses fatores, destacam-se os clínicos e sociodemográficos^(5)^.

Entre os fatores clínicos, o tempo de atendimento é determinante para o prognóstico. As terapias são tempo-dependentes, e a trombólise, quando administrada em até 4,5 horas após o início dos sintomas, reduz a mortalidade e o nível de incapacidade^(6)^. Além disso, a gravidade inicial do AVC, medida pela *National Institute of Health Stroke Scale* (NIHSS), está associada ao grau de incapacidade funcional, sendo escores mais altos indicativos de maior comprometimento neurológico^(7)^. Outros fatores, como presença de diabetes, hipertensão arterial, consumo excessivo de álcool, sedentarismo, fibrilação atrial e outras doenças cardíacas e pulmonares conferem um risco aumentado de incapacidade funcional^(8)^. Há evidências de que a COVID-19 pode interferir na resposta imunológica, provocando inflamação sistêmica, o que pode agravar a lesão cerebral após um AVCi e, consequentemente, aumentar o grau de incapacidade^(9)^.

Adicionalmente, pode influenciar o prognóstico funcional o tempo desde o início do AVCi, sendo a recuperação mais significativa nas primeiras semanas e entre três e seis meses após o evento^(10)^. O início precoce da reabilitação contribui para a redução dos danos cerebrais e melhora a funcionalidade. Além disso, a hospitalização em Unidades de AVC (UAVC), que contam com estrutura adequada e equipe especializada, está associada à menor mortalidade e dependência funcional, proporcionando melhores desfechos na alta hospitalar^(11)^. Cabe ainda ressaltar a importância da profilaxia secundária, que inclui o uso de terapia antiplaquetária para prevenir a formação de coágulos e reduzir o risco de recorrência^(12)^. Em casos de fibrilação atrial, utilizam- se anticoagulantes, enquanto as estatinas são indicadas para controle da dislipidemia, prevenção de novos eventos e melhora dos desfechos em longo prazo^(13)^. O controle da hipertensão arterial também é essencial, sendo realizado por meio de terapia medicamentosa específica^(14)^.

Dentre os fatores sociodemográficos relacionados a incapacidade funcional por AVCi destacam-se o aumento da idade, sexo feminino e viver com companheiro^(15,16,17)^. Alguns indicadores de desigualdade social como raça/cor autodeclarada negra, baixa escolaridade e renda^(17)^ também são apontados como preditores.

Apesar do conhecimento dos fatores supracitados associados a incapacidade funcional, a análise desses preditores ainda é incipiente, especialmente no Brasil, incluindo diferentes tempos de *follow up*. Além disso, a mortalidade e a incapacidade funcional por AVCi, especialmente em países em desenvolvimento como o Brasil e em regiões do país como o Nordeste, com indicadores socioeconômicos desfavoráveis, carecem de registro em curto e longo prazo.

Conhecer o grau de incapacidade e seus preditores, ao longo do tempo após o AVCi, proporciona informações relevantes para melhorar a gestão da atenção à saúde, direcionar o tratamento, as práticas de cuidados em enfermagem e as políticas públicas direcionadas às pessoas com AVCi. Além disso, pode direcionar a equipe de saúde nas ações de promoção à saúde, prevenção, controle e reabilitação do agravo.

Com base no exposto, o presente estudo objetivou analisar os fatores clínicos e sociodemográficos associados a incapacidade funcional de pessoas com AVCi entre dois e três anos após o ictus.

## MÉTODO

### Delineamento do Estudo

Trata-se de um estudo de coorte prospectiva e analítico^(18)^.

### Local do Estudo

A pesquisa foi realizada em um Hospital Geral público do estado da Bahia, com 640 leitos, de alta complexidade, terciário e de caráter assistencial.

### Amostra

O presente estudo integra o Projeto Matriz “Fatores associados a incapacidade e mortalidade por Acidente Vascular Cerebral Isquêmico e aos tempos de acesso ao tratamento.

No período do baseline do estudo foram internadas no local de estudo 320 pessoas com AVCi, às quais foram aplicados os critérios de inclusão (diagnóstico de AVCi agudo confirmado clinicamente pelo médico e registrado em prontuário com exame de imagem compatível e idade ≥ 18 anos) e os critérios de exclusão (sinais e sintomas que impossibilitassem a comunicação verbal pelo participante na ausência de acompanhante para responder aos questionamentos da pesquisa e tempo de ictus superior a 10 dias devido a possibilidade de viés recordatório). Dos 320 participantes recrutados, 12 atenderam aos critérios de exclusão, perfazendo uma amostra de 308 acompanhada por meio de visitas diárias durante a internação e, posteriormente, por ligação telefônica até 90 dias após o ictus. Dos 308 participantes, 38 vieram a óbito no hospital e 20 após a alta. Nove foram perdidos no seguimento, totalizando 242 sobreviventes em 90 dias do evento, os quais foram recontactados por telefone entre dois e três anos após o ictus, no período de março de 2021 a novembro de 2023. Dentre eles, 33 faleceram, 54 foram perdas de seguimento e 155 constituíram a amostra do presente estudo. Para esse estudo, o óbito foi critério de exclusão.

### Instrumentos de Coleta de Dados

Utilizou-se um instrumento para caracterização sociodemográfica e clínica e para a identificação do tempo de chegada ao hospital de referência em neurologia (TCRH) em horas.

Um protocolo de ligação telefônica foi também utilizado, contendo itens para registro da identificação do participante, da pontuação obtida pela aplicação da Escala de Rankin modificada (ERm), da ocorrência de óbito, novo AVC e Covid-19, do autorrelato de uso de medicação para profilaxia secundária, do seguimento em consultas com fisioterapeuta, médico, enfermeiro ou outros profissionais da saúde.

A ERm é amplamente utilizada na prática clínica e em pesquisas para avaliar o grau de incapacidade funcional relacionada às atividades de vida diária, com ênfase no comprometimento motor, face ao AVC. A versão utilizada foi a traduzida e adaptada culturalmente para o Brasil e validada para aplicação por telefone^(19)^. O grau de incapacidade é classificado numa escala de 0 a 6 pontos, a saber: 0 = assintomático, sem incapacidade; 1 = nenhuma incapacidade significativa; 2 = incapacidade leve; 3 = incapacidade moderada; 4 = incapacidade moderadamente severa, 5 = incapacidade grave e 6 = óbito^(19)^.

### Procedimentos de Coleta de Dados

Os dados sociodemográficos e clínicos foram obtidos por entrevista, exceto a NIHSS admissional, a presença de fibrilação atrial (FA), diabetes mellitus (DM), hipertensão arterial (HAS), dislipidemia e realização de trombólise, que foram levantados no prontuário. Nas situações em que o participante elegível não tinha condições clínicas, cognitivas e/ou emocionais de interagir com a pesquisadora a abordagem foi feita com o acompanhante. A data e hora do início dos sintomas ou se houve *Wake Up Stroke*, foram questionados à pessoa acometida e/ou acompanhante no momento da entrevista. A data e hora de chegada ao local do estudo foram observados no prontuário.

As ligações telefônicas entre dois e três anos do ictus foram realizadas com a utilização de smartphones, por duas graduandas e uma mestranda, as quais foram devidamente treinadas por pesquisadora do projeto que realizou as ligações de acompanhamento em até 90 dias do ictus. Nessa fase, as respostas aos instrumentos utilizados foram dadas, preferencialmente, pelo próprio participante e, na sua impossibilidade devido a condições prejudicadas de comunicação e compreensão, pela pessoa que o acompanhava nas atividades cotidianas. Três tentativas de ligação foram feitas em dias e horários diferentes da semana. Caso uma das contactantes não obtivesse êxito, o instrumento era entregue a outra para que mais duas novas tentativas fossem realizadas para se evitar ao máximo o número de perdas de participantes do estudo. Em caso de insucesso no contato telefônico, após as cinco tentativas, considerou-se perda de seguimento.

Todo participante possuía um protocolo de ligação telefônica impresso, que ficava na mão das entrevistadoras no ato da ligação, o qual continha também seu número correspondente ao banco de dados. Após registro dos dados levantados em protocolo físico, todos foram transferidos para o banco de dados no SPSS versão 22.0 e os protocolos físicos foram devidamente guardados para eventuais consultas.

### Análise de Dados

As variáveis independentes (sociodemográficas e clínicas) e a variável dependente (incapacidade funcional) foram analisadas em frequências absolutas e relativas. Para as análises bivariada e multivariada, a variável incapacidade funcional foi dicotomizada em: 0 a 2 (assintomático a incapacidade leve) e 3 a 5 (incapacidade moderada a grave). Para verificar a associação entre a incapacidade funcional e as variáveis de interesse foi empregado o teste Qui-quadrado de Pearson ou Exato de Fisher. A seguir, as variáveis cujas associações mostraram valor de p ≥ 20 foram para a análise multivariada. Considerando a proporção elevada de incapacidade severa a moderada na amostra, corrigiu-se a superestimação da *Odds Ratio*, mediante o cálculo da estimativa de RP e de seu respectivo intervalo de confiança de 95%, utilizando-se o modelo de regressão de Poisson robusto.

A modelagem foi realizada com o procedimento backward. Para escolha do melhor modelo, foi utilizado o Critério de Informação de Akaike (AIC) e escolhido aquele que apresentou menor valor. Nessa fase, a significância estatística adotada foi de 5%. Os dados foram processados e analisados no programa *Statistical Package for the Social Sciences* (SPSS), versão 22 e no STATA versão 12.

### Aspectos Éticos

O estudo integra o Projeto Matriz e está aprovado pelo Comitê de Ética da instituição, com pareceres de número 3.159.694 e 6.666.572. Aos participantes foram assegurados o sigilo da identidade pessoal, a privacidade e o direito à desistência da pesquisa em qualquer etapa, o esclarecimento dos objetivos e procedimentos do estudo e de dúvidas quanto ao conteúdo do Termo de Consentimento Livre e Esclarecido.

## RESULTADOS

Na amostra de 155 participantes, observou-se que 62,6% eram assintomáticos ou apresentavam incapacidade leve, enquanto 37,4% apresentavam incapacidade moderada a grave. Com relação às variáveis sociodemográficas, predominaram mulheres (50,3%), faixa etária 60 a 79 anos (55,5%), estado civil casado/com companheiro (52,3%), escolaridade fundamental incompleto/completo (49,0%), situação laboral inativa (73,5%), renda familiar mensal de até um salário-mínimo (45,0%) e raça-cor autodeclarada parda (55,2%). Observou-se maior percentual de mulheres com incapacidade moderada a grave (p = 0,003) e maior percentual de participantes sem atividade laboral apresentando maior incapacidade (p = 0,013). As demais variáveis sociodemográficas foram independentes do grau de incapacidade (Tabela 1).

**Tabela 1:** Associação entre variáveis sociodemográficas e grau de incapacidade funcional entre dois e três após o ictus – Salvador, Bahia, Brasil, 2023.

Variáveis sociodemográficas	Total n (%)	Grau de incapacidade funcional		Valor de p
		Rankin 0–2 n (%)	Rankin 3–5 n (%)	
Sexo				
Feminino	78 (50,3)	40 (51,3)	38 (48,7)	0,003*
Masculino	77 (49,7)	57 (74,0)	20 (26,0)	
Idade				
Até 59 anos	53 (34,2)	32 (60,4)	21 (39,6)	0,429**
60 a 79 anos	86 (55,5)	57 (63,3)	29 (33,7)	
≥ 80 anos	16 (10,3)	8 (50,0)	8 (50,0)	
Estado civil				
Casado/com companheiro	81 (52,3)	52 (64,2)	29 (32,8)	0,663**
Solteiro/viúvo/separado/Divorciado	74 (47,7)	45 (60,8)	29 (39,2)	
Escolaridade				
Superior completo ou incompleto	10 (6,5)	6 (60,0)	4 (40,0)	0,988**
Ensino médio completo ou incompleto	48 (31,0)	31 (64,6)	17 (35,4)	
Ensino Fundamental completo ou incompleto	76 (49,0)	47 (61,8)	29 (38,2)	
Analfabeto/assina o nome	21 (13,5)	13 (61,9)	8 (38,1)	
Situação laboral				
Com atividade	68 (43,9)	50 (73,5)	18 (26,5)	0,013*
Sem atividade	87 (56,1)	47 (54,0)	40 (46,0)	
Renda familiar em salário-mínimo (n = 149)				
> 3	19 (12,8)	14 (73,7)	5 (26,3)	0,517*
> 1 até 3	63 (42,2)	38 (60,3)	25 (39,7)	
Até um	67 (45,0)	40 (59,7)	27 (40,3)	
Raça/cor (n = 154)				
Branca	22 (14,3)	14 (63,6)	8 (36,4)	0,570**
Preta	47 (30,5)	32 (68,1)	15 (31,9)	
Parda	85 (55,2)	50 (58,8)	35 (41,2)	

* Valor de p obtido pelo Qui-quadrado de Pearson

**Valor de p obtido pelo teste Exato de Fisher.

Quanto às variáveis clínicas, 45,4% apresentaram NIHSS característico de déficit neurológico moderado e 35,4% déficit leve, 59,4% chegaram ao local de estudo em até 4,5 h do início dos sintomas ou *wake up stroke*, 72,9% não realizaram trombólise, 76,1% tinham HAS, 73,4% não tinham AVC prévio (73,4%) e FA (92,6%). Além disso, a maioria não fumava (60,6%), não apresentava diabetes (76,6%, dislipidemia (69,1%), infarto do miocárdio (91,0%), COVID-19 (91,6%) e novo AVC entre dois e três anos após o ictus (92,3%) (Tabela 2). Observou-se que as maiores pontuações na NIHSS (p = 0,000), o AVC prévio (p = 0,036) e a ocorrência de novo AVC entre dois e três anos do último evento (p = 0,001) foram associados a incapacidade moderada a grave. As variáveis DM, dislipidemia, IAM, tabagismo, FA, COVID-19, TCHR e submissão a trombólise foram independentes da incapacidade.

**Tabela 2:** Associação entre variáveis clínicas e incapacidade funcional entre dois e três anos do ictus – Salvador, Bahia, Brasil, 2023.

Variáveis clínicas	Total n (%)	Grau de incapacidade funcional		Valor de p*
		Rankin 0–2 n (%)	Rankin 3–5 n (%)	
NIHSS (n = 130)				
≤5	46 (35,4)	36 (78,3)	10 (21,7)	0,000
6 a 13	59 (45,4)	37 (62,7)	22 (37,3)	
≥14	25 (19,2)	7 (28,0)	18 (72,0)	
Admissão na UAVC				
Sim	120 (77,4)	75 (62,5)	45(37,5)	0,969
Não	35 (22,6)	22 (62,9)	13 (37,1)	
TCHR				
≤ 4,5 h	92 (59,4)	59 (64,1)	33 (35,9)	0,630
> 4,5 h	63 (40,6)	38 (60,3)	25 (39,7)	
Trombólise				
Sim	42 (27,1)	31 (73,8)	11 (26,2)	0,078
Não	113 (72,9)	66 (58,4)	47 (41,6)	
AVC prévio (n = 154)				
Não	113 (73,4)	76 (67,3)	37 (32,7)	0,036
Sim	41 (26,6)	20 (48,8)	21 (51,2)	
Fibrilação atrial (n = 148)				
Não	137 (92,6)	86 (62,8)	51 (37,2)	0,588
Sim	11 (7,4)	6 (54,5)	5 (45,5)	
Diabetes Mellitus (n = 154)				
Não	118 (76,6)	77 (65,3)	41 (34,7)	0,291
Sim	36 (23,4)	20 (55,6)	16 (44,4)	
Hipertensão arterial				
Não	37 (23,9)	28 (75,7)	9 (24,3)	0,059
Sim	118 (76,1)	69 (58,5)	49 (41,5)	
Dislipidemia				
Não	107 (69,1)	70 (65,4)	37 (34,6)	0,275
Sim	48 (30,9)	27 (56,3)	21 (43,8)	
Infarto do Miocárdio				
Não	141 (91,0)	89 (63,1)	52 (36,9)	0,659
Sim	14 (9,0)	8 (57,1)	6 (42,9)	
Tabagismo				
Não	94 (60,6)	61 (64,9)	33 (35,1)	0,491**
Sim	16 (10,4)	11 (68,8)	5 (31,3)	
Ex-tabagista	45 (29,0)	25 (55,6)	20 (44,4)	
COVID-19 (n = 154)				
Não	141 (91,6)	89 (63,1)	52 (36,9)	0,595
Sim	13 (8,4)	7 (53,8)	6 (46,2)	
Novo AVC				
Não	143 (92,3)	95 (66,4)	48 (33,6)	0,001*
Sim	12 (7,7)	2 (16,7)	10 (83,3)	
Profilaxia secundária (n = 132)				
Sim	83 (53,6)	54 (65,1)	29 (34,9)	0,783*
Não	49 (31,6)	29 (59,2)	20 (40,8)	
Não sabe	23 (14,8)	14 (60,9)	9 (39,1)	

* Valor de p obtido pelo Qui-quadrado de Pearson

**Valor de p obtido pelo teste Exato de Fisher.

Nas análises bivariadas, as variáveis que apresentaram diferença estatisticamente significante até 20% (sexo, atividade laboral, NIHSS, trombólise, AVC prévio, HAS e novo AVC) foram testadas como preditoras para o grau de incapacidade na análise multivariada.

O modelo final mostrou que participantes com pontuação na NIHSS de 6 a 13 e ≥14 pontos apresentaram, respectivamente 2,4 vezes (RP 2,35, IC 95% 13–4,05) e 4,4 vezes mais incapacidade moderada a grave (RP 4,40, IC 95%: 2,09–6,83) comparados aqueles com pontuação de 0 a 5. Aqueles não submetidos a trombólise e que sofreram novo AVC entre dois e três anos após o ictus apresentaram, respectivamente, 3,0 vezes (RP = 3,02, IC 95% 1,32–3,74) e 3,8 vezes (RP 3,82, IC 95% 1,49–3,47) mais incapacidade moderada a grave comparados àqueles que receberam a terapia trombolítica e sofreram novo evento (Tabela 3).

**Tabela 3:** Análise multivariada com as variáveis associadas a incapacidade funcional entre dois e três anos após o ictus – Salvador, Bahia, Brasil, 2023.

Variáveis	RP (IC 95%)
NIHSS	
≤5	1,00
6 a 13	2,35 (1,13–4,05)
≥14	4,40 (2,09–6,83)
Trombólise	
Sim	1,0
Não	3,02 (1,32–3,74)
Novo AVC	
Não	1,0
Sim	3,82 (1,49–3,47)
Critério de Informação de Akaike = 185.3753	

Os resultados mostraram que, entre dois e três anos após o AVCi, a maioria dos participantes estava assintomática ou apresentava disfunção leve e que fatores clínicos como maior déficit neurológico inicial, ausência de trombólise e a recorrência do AVCi foram os que mais contribuíram para a incapacidade funcional moderada a grave.

## DISCUSSÃO

O estudo mostrou o impacto do AVCi na vida das pessoas acometidas, pois mais de um terço apresentou incapacidade moderada a grave entre dois e três anos após o ictus. De modo geral, as pessoas com maior grau de incapacidade têm maior dificuldade para realizar tarefas da vida diária de forma independente e requerem assistência de cuidadores ou de algum tipo de equipamento que auxilie no desempenho das atividades, além de necessitarem do suporte de uma equipe multiprofissional^(20)^.

Um estudo realizado em Xangai, China (n = 305), avaliou a incapacidade funcional após AVCi por meio da Escala de Rankin modificada (ERm), e observou uma maior proporção de indivíduos assintomáticos ou com incapacidade leve entre um mês e dois anos após o evento (83,2%). Apenas 16,8% apresentavam incapacidade moderada a severa, percentual inferior ao identificado no presente estudo. No estudo chinês, graus mais baixos de incapacidade funcional foram associados ao comprometimento cognitivo, à presença de sintomas depressivos e ao histórico prévio de AVC — dado que, embora não tenha apresentado significância estatística em nossa amostra, pode estar relacionado a piores desfechos^(21)^.

A amostra foi majoritariamente constituída por pessoas idosas e idosas longevas que têm maior chance de AVCi ao conviverem com doenças que afetam o sistema cardiovascular. Estima-se que o risco de AVCi duplique a cada 10 anos, a partir dos 55 anos^(22)^. Nossos resultados corroboram outro estudo^(21)^, mostrando que 77,1% tiveram AVCi entre 61 e 80 anos. Apesar da independência entre idade e incapacidade, ressalta-se a importância da prevenção e do controle dos fatores de risco para o evento em todas as fases da vida.

O AVCi afetou proporções semelhantes entre os sexos, com predomínio dos casos em mulheres, achado que corrobora dados de um estudo nacional^(15)^. Esse maior acometimento no sexo feminino pode estar relacionado a níveis mais elevados de lipídios, à menopausa e ao impacto do envelhecimento sobre a saúde vascular. No presente estudo, as mulheres apresentaram, em maior proporção, incapacidade moderada a grave, e possuem maior probabilidade de evolução para um prognóstico desfavorável, com risco aumentado de óbito^(23)^.

A maioria dos participantes do estudo vivia com um companheiro. Considerando o elevado percentual de indivíduos com incapacidade moderada a grave, observa-se uma dependência significativa de apoio social e auxílio no autocuidado. Pessoas casadas ou em união estável tendem a receber mais suporte emocional e prático de seus cônjuges durante a recuperação pós-AVC isquêmico, o que pode favorecer a adesão às recomendações médicas e a adoção de um estilo de vida saudável, contribuindo para um prognóstico mais favorável^(16)^. No entanto, no presente estudo, as variáveis estado civil e grau de incapacidade mostraram-se independentes.

É válido destacar que o enfermeiro pode identificar os cônjuges de vítimas de AVCi que podem oferecer suporte e serem ativos no planejamento, na supervisão e na execução de cuidados no domicílio, assim como na providência de recursos, na motivação e no acompanhamento do processo de reabilitação. Em situação de desigualdade social, como se encontrava a maioria da amostra, o desafio para o cuidado após o AVCi é ainda maior. A amostra foi, predominantemente, formada por pessoas negras e pardas, com baixa escolaridade, baixa renda e sem atividade laboral. A baixa escolaridade pode resultar em baixos rendimentos, menor conhecimento sobre os fatores de risco e, consequentemente, menor prevenção e controle da doença^(17)^. As pessoas brancas, em geral, têm maior acesso à reabilitação após o AVCi comparadas às pretas e pardas^(17)^. Em nosso estudo, apesar da ausência de significância estatística entre renda e incapacidade, maior percentual dos participantes com menor poder econômico foi identificado com incapacidade grave. Assim, é necessário assegurar, no processo de reabilitação, o acesso contínuo a serviços públicos para os grupos em situação de hipossuficiência socioeconômica, a fim de assegurar a efetividade de sua recuperação e inclusão social.

Os participantes sem atividade laboral apresentaram incapacidade moderada a grave em maior proporção. Um estudo realizado com japoneses mostrou risco aumentado para morbimortalidade por AVC entre aqueles que passaram pelo menos um período sem exercer atividade laboral ou tiveram empregos perdidos em comparação aos ativos no trabalho^(24)^. É possível aventar a hipótese de que a situação laboral ativa propicie o estímulo físico e cognitivo com recuperação de habilidades e melhora do estado emocional.

Referente às variáveis clínicas, um percentual elevado de participantes tinha hipertensão arterial, um fator de risco independente para o AVC e a sua recorrência. Esses apresentaram em maior proporção incapacidade moderada a grave, com diferença estatística *borderline* na análise bivariada. A hipertensão arterial está associada a pior prognóstico funcional, por aumentar o risco de edema cerebral e hemorragia intracerebral. Indivíduos hipertensos acometidos por AVCi tendem a apresentar maior gravidade do déficit neurológico inicial, avaliado pela escala NIHSS, além de maior extensão da lesão cerebral e taxas mais elevadas de incapacidade funcional em comparação aos normotensos^(25)^.

Uma menor proporção de participantes apresentou fibrilação atrial, enquanto o diabetes mellitus acometeu cerca de um quarto da amostra e a dislipidemia, aproximadamente um terço. Essas condições clínicas estão associadas a maior risco de morbimortalidade por AVCi, por potencializarem danos micro e macrovasculares^(26)^, e exigirem manejo clínico contínuo. No entanto, no presente estudo, tais variáveis mostraram-se independentes do grau de incapacidade funcional. Ressalta-se a importância do diagnóstico preciso de fibrilação atrial após o AVCi, uma vez que permite a implementação de estratégias específicas de prevenção secundária, como o uso de anticoagulantes, que contribuem para a redução do risco de recorrência^(27)^.

Durante o período de seguimento, alguns participantes foram diagnosticados com COVID-19; no entanto, não foi observada associação entre a infecção e o grau de incapacidade funcional. Ainda assim, a literatura aponta que indivíduos que contraem COVID-19 após um AVC apresentam maior propensão à recorrência do evento, formas mais graves da doença e complicações cardiovasculares^(28)^.

Aproximadamente um terço da amostra apresentou histórico de AVC, o qual foi associado a maior grau de incapacidade apenas na análise bivariada. Por outro lado, a recorrência do AVCi durante o período de seguimento foi uma das três variáveis significativamente associadas à incapacidade moderada a grave na análise multivariada, aumentando em 3,82 vezes a chance de disfunção funcional. Corroborando esses achados, Yang e colaboradores^(29)^ demonstraram, por meio de regressão logística múltipla, que a recidiva do AVC é preditora de maior grau de incapacidade. O AVCi é responsável por incapacidades agudas que tendem a se estabilizar após um período de três a seis meses. No entanto, a estabilização é prejudicada caso novos eventos ocorram.

A história prévia de AVC e a sua recorrência, observada em nosso estudo, podem levar ao agravamento de déficits relacionados à marcha e ao comprometimento da função motora devido a novas lesões cerebrais^(29)^. A recorrência do AVCi em até dois e três anos após o ictus evidenciou as dificuldades na prevenção e controle da doença e a importância de seguimento continuado das pessoas acometidas com equipe multiprofissional, avaliação e suporte para adesão a terapêutica. Uma coorte prospectiva com 764 pacientes observou que 18,0% tiveram um novo AVCi nos três primeiros anos após a alta, corroborando nossos resultados, sendo a falta de adesão à profilaxia secundária, após o primeiro evento, um fator de risco para a recorrência^(12)^.

Na presente investigação, o déficit neurológico moderado avaliado pela NIHSS foi mais frequente. Na escala, à medida que a pontuação aumenta, expressa maior comprometimento neurológico e incapacidade, o que se traduz em resultados desfavoráveis para a qualidade de vida^(16)^. Na análise bivariada, a incapacidade moderada a grave foi proporcionalmente crescente em razão da gravidade do evento. A análise multivariada tornou mais evidente a relação estabelecida entre a gravidade do déficit neurológico e o grau de incapacidade, pois as pessoas com déficit neurológico moderado e grave tiveram, respectivamente, 2,35 e 4,40 vezes mais incapacidade moderada a grave comparadas àquelas com incapacidade leve. Uma coorte de cinco anos avaliou pessoas com e sem incapacidade funcional após um AVCi, mostrando que 45% da amostra (n = 893) permaneceram com incapacidade funcional após esse período e que o NIHSS mais alto foi significantemente associado a maior grau de incapacidade, corroborando nossos achados^(29)^. Outro estudo, realizado com 101 pessoas que sofreram AVCi, avaliadas em um, três e seis meses do evento, observou que após seis meses do ictus 50% não conseguiram recuperar suas funções e tinham pontuações mais altas no NIHSS^(16)^.

A maioria dos participantes chegou dentro da janela terapêutica ao local do estudo; no entanto, menos de 30% foram submetidos a trombólise, o que contribuiu para maior grau de incapacidade, como mostrou a análise multivariada. Os participantes não submetidos à trombólise tiveram 3,0 vezes mais incapacidade moderada a grave em comparação àqueles que receberam o tratamento trombolítico, evidenciando os seus benefícios em dois e três anos do evento. Ademais, um percentual expressivo chegou fora da janela terapêutica deixando de usufruir dos seus benefícios. Atualmente, as diretrizes para o AVCi limitam o início da terapia trombolítica intravenosa a 4,5 horas após o início dos sintomas ou *wake up stroke*. A utilização da terapia com alteplase mostra que as pessoas beneficiadas apresentaram déficits neurológicos mínimos ou ausentes^(1)^. Outros autores reforçaram os efeitos positivos da trombólise, ajudando a reduzir a incapacidade, aumentando a probabilidade de retorno mais rápido ao trabalho e promovendo a melhora da qualidade de vida^(30)^.

Reforça-se a importância da atuação do enfermeiro no apoio ao paciente e à família desde o início do processo de reabilitação, especialmente na fase de transição do hospital para o domicílio. Nesse contexto, cabe ao profissional identificar os potenciais cuidadores e capacitá-los para oferecer suporte adequado ao paciente. Esse processo requer adaptação tanto por parte do paciente quanto da família, sobretudo nos casos de maior grau de incapacidade. As orientações compartilhadas pelo enfermeiro ainda no ambiente hospitalar são fundamentais não apenas para o cuidado domiciliar, mas também para assegurar a continuidade do acompanhamento na atenção básica, promovendo a vinculação com a unidade de saúde^(31)^.

Os resultados do presente estudo indicaram que maior déficit neurológico, ausência de trombólise e recorrência do AVCi foram os principais fatores associados à incapacidade moderada a grave. Esses achados ressaltam a importância de políticas públicas voltadas ao cuidado de pessoas acometidas por AVCi, bem como de estratégias efetivas de reabilitação em todas as fases do tratamento, com o objetivo de minimizar o grau de incapacidade funcional. Reforça-se a necessidade de estudos voltados ao acompanhamento longitudinal desses indivíduos por equipes multiprofissionais no sistema público de saúde, assegurando o acesso universal à reabilitação de qualidade e a ações eficazes de prevenção secundária.

Os resultados reforçam a importância da adesão a estratégias consolidadas de prevenção, como a iniciativa HEARTS, que representa um elo fundamental na prevenção de doenças cerebrovasculares. Trata-se de um acrônimo que engloba seis pilares essenciais: hábitos saudáveis; protocolos clínicos baseados em evidências; acesso a medicamentos, tecnologias e insumos; manejo do risco cardiovascular; atuação em equipe multidisciplinar; e monitoramento por meio de indicadores padronizados. A implementação efetiva dessa estratégia pode contribuir significativamente para melhores resultados na prevenção e controle do AVCi.

Os achados desta pesquisa também evidenciam a necessidade de ampliação da cobertura do sistema público de saúde no que se refere ao uso da terapêutica trombolítica, eficaz na redução de disfunções neurológicas mais graves. Paralelamente, é fundamental que a comunidade e os indivíduos acometidos sejam orientados a reconhecer precocemente os sinais e sintomas do AVC isquêmico, a fim de buscar atendimento em unidades de referência em neurologia dentro da janela terapêutica. No presente estudo, aproximadamente 40% dos participantes chegaram ao serviço hospitalar fora da janela terapêutica. Aqui, reside um espaço fundamental para a atuação do enfermeiro por meio de atividades de educação em saúde. Além disso, um país com menos disparidades sociais seria capaz de assegurar melhores condições de vida minimizando o adoecimento e o sofrimento decorrente.

Este estudo apresenta algumas limitações que devem ser consideradas na interpretação dos resultados. Houve uma perda de participantes durante o seguimento, o que pode comprometer a representatividade da amostra final. Além disso, a coleta de dados por meio de entrevistas telefônicas e, em alguns casos, em que os dados foram fornecidos por cuidadores e familiares, pode ter gerado algum viés de informação. Outro viés a ser considerado refere-se à seleção dos participantes no baseline, uma vez que alguns pacientes em condições mais graves ou com déficits cognitivos sem acompanhantes disponíveis no momento da inclusão foram excluídos, o que pode limitar a generalização dos achados para a população com maior comprometimento funcional.

## CONCLUSÃO

O estudo mostrou o impacto do AVCi na vida das pessoas acometidas, com mais de um terço com incapacidade moderada a grave entre dois e três anos após ictus. As variáveis associadas a incapacidade moderada a severa, na análise multivariada, foram maior gravidade do déficit neurológico, não realização de trombólise e recorrência do AVCi. O estudo mostra a necessidade de avanços na rede de atenção à saúde, assegurando a ampliação da oferta de trombólise, o acesso a unidades de tratamento especializadas e a reabilitação precoce e continuada, com equipe multiprofissional. Salienta que são fundamentais as políticas e intervenções efetivas voltadas à promoção da saúde, a prevenção e controle dos fatores de risco para o AVCi, além do estímulo à apresentação precoce das vítimas a um hospital de referência em neurologia para minimizar as repercussões negativas do AVCi na vida pessoal, familiar e para a sociedade em geral.

## Data Availability

O conjunto de dados que dá suporte aos resultados deste estudo não está disponível publicamente.

# Translation: Predictores de incapacidad funcional en personas con accidente cerebrovascular isquémico: dos a tres años después del ACV*

**DOI:** 10.1590/1980-220X-REEUSP-2025-0150es

**Authors:** Brenda Silva Cunha, Mariana de Almeida Moraes, Liane de Assis Campos Medeiros, Ludimila Santos Muniz, Carlos Antônio de Souza Teles Santos, Maria Cecília Bueno Jayme Gallani, Fernanda Carneiro Mussi

**Affiliations:** 1 Universidade Federal da Bahia, Escola de Enfermagem, Salvador, BA, Brasil.; 2 Université Laval, Quebec, QC, Canada.

**Keywords:** Accidente Cerebrovascular Isquémico, Personas con Discapacidad, Terapia Trombolítica, Rehabilitación, Cuidadores, Enfermería

## Abstract

**Objetivo:**

Analizar los factores clínicos y sociodemográficos asociados a la discapacidad funcional en personas con accidente cerebrovascular isquémico (ACVi) entre 2 y 3 años después del evento.

**Método:**

Cohorte prospectiva con 241 participantes. Se utilizaron instrumentos para la caracterización sociodemográfica y clínica, la Escala de Rankin-m y un protocolo de llamada telefónica. Los datos se analizaron con pruebas de Qui-cuadrado de Pearson o Exacta de Fisher y el modelo de Poisson robusto, con un nivel de significación del 5%.

**Resultados:**

De los participantes, el 62,6% presentó una puntuación de Rankin de 0 a 2. El análisis multivariado mostró que un mayor déficit neurológico (6–13 y ≥14) se asoció 2,35 (IC 95% 1,13; 4,05) y 4,4 (IC 95% 2,09; 6,83) veces más con discapacidad moderada a grave; la no realización de trombólisis y la recurrencia del ACVi fueron, respectivamente, 3,02 (IC 95%: 1,32; 3,74) y 3,82 veces (IC 95%: 1,49; 3,47) más asociadas a la incapacidad moderada a grave.

**Conclusión:**

El evento afectó a la capacidad funcional, con un mayor déficit neurológico, la no realización de trombólisis y la recurrencia del ACV como principales predictores de discapacidad. Estrategias como la ampliación de la trombólisis, la atención precoz, la rehabilitación continua y el control de los factores de riesgo pueden reducir las discapacidades.

## INTRODUCCIÓN

El accidente cerebrovascular (ACV) es una de las principales causas de muerte, hospitalización y discapacidad adquirida en todo el mundo. Los tipos isquémico (ACVi) y hemorrágico se consideran emergencias clínicas, siendo el ACVi responsable de alrededor del 85% de los casos^(1)^.

En Brasil, el ACV fue la segunda causa principal de muerte en los años 1990 y 2019. Se estima que una de cada cuatro personas sufrirá un ACV a lo largo de su vida y que cada año se producen alrededor de 17 millones de casos en el país, de los cuales aproximadamente un tercio evoluciona hacia la muerte^(2)^. Además, las tasas de hospitalización superaron las 185 000 en 2022, lo que generó elevados gastos para el Sistema Único de Salud^(1)^. Un estudio de la *Lancet Neurology Commission* ^(3)^ prevé que, para 2050, las muertes podrían aumentar en un 50%, principalmente en los países de ingresos bajos y medios.

El ACV es la principal causa de discapacidad a largo plazo, lo que compromete la integración social de las personas. Este impacto puede llevar a la jubilación anticipada, afectando a alrededor del 40% de los afectados. Se estima que aproximadamente el 70% de las personas que han sufrido un accidente cerebrovascular no pueden reanudar sus actividades profesionales, y alrededor del 50% pasan a depender de cuidadores para realizar las tareas cotidianas^(4)^.

Este panorama pone de manifiesto la importancia de la promoción de la salud, la prevención y el control de los factores de riesgo de accidente cerebrovascular, así como el acceso rápido y adecuado al tratamiento en todos los niveles de atención. También es fundamental comprender los factores intrínsecos y extrínsecos que influyen en el grado de discapacidad, con el objetivo de minimizarlos. Entre estos factores, destacan los clínicos y sociodemográficos^(5)^.

Entre los factores clínicos, el tiempo de atención es determinante para el pronóstico. Las terapias dependen del tiempo, y la trombólisis, cuando se administra en un plazo de 4,5 horas tras el inicio de los síntomas, reduce la mortalidad y el nivel de discapacidad^(6)^. Además, la gravedad inicial del ACV, medida por la *National Institute of Health Stroke Scale* (NIHSS), está asociada al grado de discapacidad funcional, siendo las puntuaciones más altas indicativas de un mayor compromiso neurológico^(7)^. Otros factores, como la presencia de diabetes, hipertensión arterial, consumo excesivo de alcohol, sedentarismo, fibrilación auricular y otras enfermedades cardíacas y pulmonares, confieren un mayor riesgo de discapacidad funcional^(8)^. Hay pruebas de que la COVID-19 puede interferir en la respuesta inmunitaria, provocando inflamación sistémica, lo que puede agravar la lesión cerebral tras un ACVi y, en consecuencia, aumentar el grado de discapacidad^(9)^.

Además, el tiempo transcurrido desde el inicio del ACVi puede influir en el pronóstico funcional, siendo la recuperación más significativa en las primeras semanas y entre tres y seis meses después del evento^(10)^. El inicio precoz de la rehabilitación contribuye a reducir el daño cerebral y mejora la funcionalidad. Además, la hospitalización en unidades de ACV (UACV), que cuentan con una estructura adecuada y personal especializado, se asocia con una menor mortalidad y dependencia funcional, lo que proporciona mejores resultados al alta hospitalaria^(11)^. Cabe destacar también la importancia de la profilaxis secundaria, que incluye el uso de terapia antiplaquetaria para prevenir la formación de coágulos y reducir el riesgo de recurrencia^(12)^. En casos de fibrilación auricular, se utilizan anticoagulantes, mientras que las estatinas están indicadas para controlar la dislipidemia, prevenir nuevos eventos y mejorar los resultados a largo plazo^(13)^. El control de la hipertensión arterial también es esencial y se lleva a cabo mediante terapia farmacológica específica^(14)^.

Entre los factores sociodemográficos relacionados con la incapacidad funcional por ACV destacan la edad avanzada, el sexo femenino y vivir con pareja^(15,16,17)^. Algunos indicadores de desigualdad social, como la raza/color autodeclarado negro, el bajo nivel educativo y los bajos ingresos^(17)^, también se señalan como predictores.

A pesar del conocimiento de los factores mencionados anteriormente asociados a la incapacidad funcional, el análisis de estos predictores aún es incipiente, especialmente en Brasil, incluyendo diferentes tiempos de seguimiento. Además, la mortalidad y la incapacidad funcional por ACVi, especialmente en países en desarrollo como Brasil y en regiones del país como el Nordeste, con indicadores socioeconómicos desfavorables, carecen de registros a corto y largo plazo.

Conocer el grado de incapacidad y sus predictores a lo largo del tiempo después del ACVi proporciona información relevante para mejorar la gestión de la atención sanitaria, orientar el tratamiento, las prácticas de cuidados de enfermería y las políticas públicas dirigidas a las personas con ACVi. Además, puede orientar al equipo sanitario en las acciones de promoción de la salud, prevención, control y rehabilitación de la enfermedad.

En base a lo anterior, el presente estudio tuvo como objetivo analizar los factores clínicos y sociodemográficos asociados a la incapacidad funcional de personas con AVCi entre dos y tres años después del ACV.

## MÉTODO

### Diseño del Estudio

Se trata de un estudio de cohorte prospectivo y analítico^(18)^.

### Lugar del Estudio

La investigación se llevó a cabo en un hospital general público del estado de Bahía, con 640 camas, de alta complejidad, terciario y de carácter asistencial.

### Muestra

El presente estudio forma parte del Proyecto Matriz «Factores asociados a la incapacidad y la mortalidad por accidente cerebrovascular isquémico y al tiempo de acceso al tratamiento».

En el periodo de referencia del estudio, ingresaron en el centro 320 personas con ACV, a las que se aplicaron los criterios de inclusión (diagnóstico de ACVi agudo confirmado clínicamente por un médico y registrado en la historia clínica con examen de imagen compatible y edad ≥ 18 años) y los criterios de exclusión (signos y síntomas que imposibilitaran la comunicación verbal por parte del participante en ausencia de un acompañante para responder a las preguntas de la investigación y tiempo de ACV superior a 10 días debido a la posibilidad de sesgo de recuerdo). De los 320 participantes reclutados, 12 cumplieron los criterios de exclusión, lo que dio una muestra de 308 acompañados mediante visitas diarias durante la hospitalización y, posteriormente, por teléfono hasta 90 días después del ACV. De los 308 participantes, 38 fallecieron en el hospital y 20 después del alta. Nueve se perdieron durante el seguimiento, lo que totalizó 242 sobrevivientes a los 90 días del evento, quienes fueron contactados nuevamente por teléfono entre dos y tres años después del ACV, en el período comprendido entre marzo de 2021 y noviembre de 2023. Entre ellos, 33 fallecieron, 54 se perdieron durante el seguimiento y 155 constituyeron la muestra del presente estudio. Para este estudio, la muerte fue criterio de exclusión.

### Instrumentos de Recolección de Datos

Se utilizó un instrumento para la caracterización sociodemográfica y clínica y para la identificación del tiempo de llegada al hospital de referencia en neurología (TCRH) en horas.

También se utilizó un protocolo de llamada telefónica que contenía ítems para registrar la identificación del participante, la puntuación obtenida mediante la aplicación de la Escala de Rankin modificada (ERm), la ocurrencia de fallecimiento, de un nuevo ACV y de Covid-19, del autoinforme sobre el uso de medicación para la profilaxis secundaria, del seguimiento en consultas con fisioterapeutas, médicos, enfermeros u otros profesionales de la salud.

La ERm se utiliza ampliamente en la práctica clínica y en investigaciones para evaluar el grado de incapacidad funcional relacionada con las actividades de la vida diaria, con énfasis en el compromiso motor, frente al ACV. La versión utilizada fue la traducida y adaptada culturalmente para Brasil y validada para su aplicación por teléfono^(19)^. El grado de discapacidad se clasifica en una escala de 0 a 6 puntos, a saber: 0 = asintomático, sin discapacidad; 1 = sin discapacidad significativa; 2 = discapacidad leve; 3 = discapacidad moderada; 4 = discapacidad moderadamente grave, 5 = discapacidad grave y 6 = muerte^(19)^.

### Procedimientos de Recolección de Datos

Los datos sociodemográficos y clínicos se obtuvieron mediante entrevista, excepto la NIHSS de admisión, la presencia de fibrilación auricular (FA), diabetes mellitus (DM), hipertensión arterial (HTA), dislipidemia y realización de trombólisis, que se recopilaron en el historial clínico. En los casos en que el participante elegible no reunía las condiciones clínicas, cognitivas y/o emocionales para interactuar con la investigadora, se abordó al acompañante. Se preguntó a la persona afectada y/o al acompañante en el momento de la entrevista la fecha y la hora de inicio de los síntomas o si se había producido un *Wake Up Stroke*. La fecha y la hora de llegada al lugar del estudio se observaron en el historial clínico.

Las llamadas telefónicas entre dos y tres años después del ACV se realizaron con teléfonos inteligentes por dos estudiantes de grado y una estudiante de maestría, quienes fueron debidamente capacitadas por la investigadora del proyecto que realizó las llamadas de seguimiento en un plazo de 90 días después del ACV. En esta fase, las respuestas a los instrumentos utilizados fueron proporcionadas, preferentemente, por el propio participante y, en caso de imposibilidad debido a condiciones que dificultaban la comunicación y la comprensión, por la persona que lo acompañaba en las actividades cotidianas. Se realizaron tres intentos de llamada en días y horarios diferentes de la semana. Si una de las personas encargadas de realizar las llamadas no lo conseguía, el instrumento se entregaba a otra para que realizara dos intentos más, con el fin de evitar en la medida de lo posible la pérdida de participantes en el estudio. Si tras cinco intentos no se conseguía contactar por teléfono, se consideraba pérdida de seguimiento.

Todos los participantes tenían un protocolo de llamada telefónica impreso, que las entrevistadoras tenían en sus manos en el momento de la llamada, y que también contenía su número correspondiente a la base de datos. Tras registrar los datos recopilados en un protocolo físico, todos fueron transferidos a la base de datos en SPSS versión 22.0 y los protocolos físicos se guardaron debidamente para futuras consultas.

### Análisis de Datos

Las variables independientes (sociodemográficas y clínicas) y la variable dependiente (incapacidad funcional) se analizaron en frecuencias absolutas y relativas. Para los análisis bivariados y multivariados, la variable incapacidad funcional se dicotomizó en: 0 a 2 (asintomático a incapacidad leve) y 3 a 5 (incapacidad moderada a grave). Para verificar la asociación entre la incapacidad funcional y las variables de interés se utilizó la prueba de chi cuadrado de Pearson o la prueba exacta de Fisher. A continuación, las variables cuyas asociaciones mostraron un valor de p ≥ 20 se sometieron a análisis multivariante. Teniendo en cuenta la elevada proporción de incapacidad grave a moderada en la muestra, se corrigió la sobreestimación de la *Odds Ratio* mediante el cálculo de la estimación de la RP y su respectivo intervalo de confianza del 95%, utilizando el modelo de regresión de Poisson robusto.

El modelado se realizó con el procedimiento *backward*. Para elegir el mejor modelo, se utilizó el Criterio de Información de Akaike (AIC) y se eligió el que presentó el valor más bajo. En esta fase, la significación estadística adoptada fue del 5%. Los datos se procesaron y analizaron en el programa *Statistical Package for the Social Sciences* (SPSS), versión 22, y en STATA, versión 12.

### Aspectos Éticos

El estudio forma parte del Proyecto Matriz y está aprobado por el Comité de Ética de la institución, con los dictámenes n.º 3.159.694 y 6.666.572. Se garantizó a los participantes el secreto de su identidad personal, la privacidad y el derecho a retirarse de la investigación en cualquier momento, la aclaración de los objetivos y procedimientos del estudio y la resolución de dudas sobre el contenido del Término de Consentimiento Libre y Esclarecido.

## RESULTADOS

En la muestra de 155 participantes, se observó que el 62,6% eran asintomáticos o presentaban una discapacidad leve, mientras que el 37,4% presentaban una discapacidad moderada a grave. En cuanto a las variables sociodemográficas, predominaron las mujeres (50,3%), el grupo de edad de 60 a 79 años (55,5%), el estado civil casado/con pareja (52,3%), educación primaria incompleta/completa (49,0%), situación laboral inactiva (73,5%), ingresos familiares mensuales de hasta un salario mínimo (45,0%) y raza-color autodeclarada como parda (55,2%). Se observó un mayor porcentaje de mujeres con discapacidad moderada a grave (p = 0,003) y un mayor porcentaje de participantes sin actividad laboral que presentaban una mayor discapacidad (p = 0,013). Las demás variables sociodemográficas fueron independientes del grado de discapacidad (Tabla 1).

**Tabla 1:** Asociación entre variables sociodemográficas y grado de incapacidad funcional entre dos y tres días después del ACV – Salvador, Bahia, Brasil, 2023.

Variables sociodemográficas	Total n (%)	Grado de incapacidad funcional		Valor de p
		Rankin 0–2 n (%)	Rankin 3–5 n (%)	
Sexo				
Mujer	78 (50,3)	40 (51,3)	38 (48,7)	0,003*
Varón	77 (49,7)	57 (74,0)	20 (26,0)	
Edad				
Hasta 59 años	53 (34,2)	32 (60,4)	21 (39,6)	0,429**
De 60 a 79 años	86 (55,5)	57 (63,3)	29 (33,7)	
≥ 80 años	16 (10,3)	8 (50,0)	8 (50,0)	
Estado civil				
Casado/con pareja	81 (52,3)	52 (64,2)	29 (32,8)	0,663**
Soltero/viudo/separado/Divorciado	74 (47,7)	45 (60,8)	29 (39,2)	
Educación				
Titulación universitaria completa o incompleta	10 (6,5)	6 (60,0)	4 (40,0)	0,988**
Bachillerato completo o incompleto	48 (31,0)	31 (64,6)	17 (35,4)	
Enseñanza primaria completa o incompleta	76 (49,0)	47 (61,8)	29 (38,2)	
Analfabetos/sabe firmar	21 (13,5)	13 (61,9)	8 (38,1)	
Situación laboral				
Trabaja	68 (43,9)	50 (73,5)	18 (26,5)	0,013*
Sin actividad	87 (56,1)	47 (54,0)	40 (46,0)	
Ingresos familiares en salario mínimo (n = 149)				
> 3	19 (12,8)	14 (73,7)	5 (26,3)	0,517*
> 1 a 3	63 (42,2)	38 (60,3)	25 (39,7)	
Hasta 1	67 (45,0)	40 (59,7)	27 (40,3)	
Raza/color (n = 154)				
Blanco	22 (14,3)	14 (63,6)	8 (36,4)	0,570**
Negro	47 (30,5)	32 (68,1)	15 (31,9)	
Marrón	85 (55,2)	50 (58,8)	35 (41,2)	

* Valor p obtenido mediante el chi-cuadrado de Pearson

** Valor p obtenido mediante la prueba exacta de Fisher.

En cuanto a las variables clínicas, el 45,4% tenía NIHSS característico de déficit neurológico moderado y el 35,4% de déficit leve, el 59,4% llegó al centro de estudio en las 4,5 horas siguientes al inicio de los síntomas o al despertar del ACV, el 72,9% no se había sometido a trombólisis, el 76,1% tenía HTA, el 73,4% no tenía ACV previo (73,4%) y FA (92,6%). Además, la mayoría no fumaba (60,6%), no tenía diabetes (76,6%), dislipidemia (69,1%), infarto de miocardio (91,0%), COVID-19 (91,6%) y un nuevo ACV entre dos y tres años después del ACV (92,3%) (Tabla 2). Las puntuaciones más altas del NIHSS (p = 0,000), el ACV previo (p = 0,036) y la aparición de un nuevo ACV entre dos y tres años después del último evento (p = 0,001) se asociaron con una discapacidad de moderada a grave. Las variables DM, dislipidemia, IAM, tabaquismo, FA, Covid-19, TCHR y someterse a trombólisis fueron independientes de la discapacidad.

**Tabla 2:** Asociación entre variables clínicas y discapacidad funcional entre dos y tres años después del ACV. Salvador, Bahia, Brasil, 2023.

Variables clínicas	Total n (%)	Grado de incapacidad funcional		Valor de p*
		Rankin 0–2 n (%)	Rankin 3–5 n (%)	
NIHSS (n = 130)				
≤5	46 (35,4)	36 (78,3)	10 (21,7)	0,000
6 a 13	59 (45,4)	37 (62,7)	22 (37,3)	
≥14	25 (19,2)	7 (28,0)	18 (72,0)	
Admisión en la UACV				
Sí	120 (77,4)	75 (62,5)	45(37,5)	0,969
No	35 (22,6)	22 (62,9)	13 (37,1)	
TCHR				
≤ 4,5 h	92 (59,4)	59 (64,1)	33 (35,9)	0,630
> 4,5 h	63 (40,6)	38 (60,3)	25 (39,7)	
Trombólisis				
Sí	42 (27,1)	31 (73,8)	11 (26,2)	0,078
No	113 (72,9)	66 (58,4)	47 (41,6)	
Accidente cerebrovascular previo (n = 154)				
No	113 (73,4)	76 (67,3)	37 (32,7)	0,036
Sí	41 (26,6)	20 (48,8)	21 (51,2)	
Fibrilación auricular (n = 148)				
No	137 (92,6)	86 (62,8)	51 (37,2)	0,588
Sí	11 (7,4)	6 (54,5)	5 (45,5)	
Diabetes mellitus (n = 154)				
No	118 (76,6)	77 (65,3)	41 (34,7)	0,291
Sí	36 (23,4)	20 (55,6)	16 (44,4)	
Hipertensión arterial				
No	37 (23,9)	28 (75,7)	9 (24,3)	0,059
Sí	118 (76,1)	69 (58,5)	49 (41,5)	
Dislipidemia				
No	107 (69,1)	70 (65,4)	37 (34,6)	0,275
Sí	48 (30,9)	27 (56,3)	21 (43,8)	
Infarto de miocardio				
No	141 (91,0)	89 (63,1)	52 (36,9)	0,659
Sí	14 (9,0)	8 (57,1)	6 (42,9)	
Tabaquismo				
No	94 (60,6)	61 (64,9)	33 (35,1)	0,491**
Sí	16 (10,4)	11 (68,8)	5 (31,3)	
Fue fumador	45 (29,0)	25 (55,6)	20 (44,4)	
COVID-19 (n = 154)				
No	141 (91,6)	89 (63,1)	52 (36,9)	0,595
Sí	13 (8,4)	7 (53,8)	6 (46,2)	
Nuevo ACV				
No	143 (92,3)	95 (66,4)	48 (33,6)	0,001*
Sí	12 (7,7)	2 (16,7)	10 (83,3)	
Profilaxis secundaria (n = 132)				
Sí	83 (53,6)	54 (65,1)	29 (34,9)	0,783*
No	49 (31,6)	29 (59,2)	20 (40,8)	
No sabe	23 (14,8)	14 (60,9)	9 (39,1)	

* p-valor obtenido por Chi-cuadrado de Pearson

** valor de p obtenido por la prueba exacta de Fisher.

En los análisis bivariantes, las variables que mostraron una diferencia estadísticamente significativa de hasta el 20% (sexo, actividad laboral, NIHSS, trombólisis, ACV previo, HTA y nuevo ACV) se probaron como predictores del grado de discapacidad en el análisis multivariante.

El modelo final mostró que los participantes con puntuaciones NIHSS de 6 a 13 y ≥14 puntos tenían 2,4 veces (RP 2,35; IC 95%: 13–4,05) y 4,4 veces más discapacidad de moderada a grave respectivamente (RP 4,40; IC 95%: 2,09–6,83) en comparación con los que tenían puntuaciones de 0 a 5. Aquellos que no se sometieron a trombolisis y que sufrieron un nuevo ACV entre dos y tres años después del ACV tuvieron, respectivamente, 3,0 veces (RP = 3,02; IC 95%: 1,32–3,74) y 3,8 veces (RP 3,82; IC 95%: 1,49–3,47) más discapacidad de moderada a grave en comparación con los que recibieron tratamiento trombolítico y sufrieron un nuevo evento (Tabla 3).

**Tabla 3:** Análisis multivariante de las variables asociadas a la discapacidad funcional entre dos y tres años después del ACV – Salvador, Bahía, Brasil, 2023.

Variables	RP (IC 95%)
NIHSS	
≤5	1,00
6 a 13	2,35 (1,13–4,05)
≥14	4,40 (2,09–6,83)
Trombólisis	
Sí	1,0
No	3,02 (1,32–3,74)
Nuevo ACV	
No	1,0
Sí	3,82 (1,49–3,47)
Criterio de información de Akaike = 185,3753	

Los resultados mostraron que, entre dos y tres años después del ACVi, la mayoría de los participantes estaban asintomáticos o presentaban una disfunción leve, y que los factores clínicos como un mayor déficit neurológico inicial, la ausencia de trombólisis y la recurrencia del ACVi fueron los que más contribuyeron a la discapacidad funcional moderada a grave.

## DISCUSIÓN

El estudio mostró el impacto del ACVi en la vida de las personas afectadas, ya que más de un tercio presentó discapacidad moderada a grave entre dos y tres años después del ACV. En general, las personas con mayor grado de discapacidad tienen más dificultades para realizar las tareas de la vida diaria de forma independiente y requieren la asistencia de cuidadores o algún tipo de equipo que les ayude a realizar las actividades, además de necesitar el apoyo de un equipo multidisciplinar^(20)^.

Un estudio realizado en Shanghái, China (n = 305), evaluó la discapacidad funcional tras un ACV mediante la Escala de Rankin modificada (ERm) y observó una mayor proporción de individuos asintomáticos o con discapacidad leve entre un mes y dos años después del evento (83,2%). Solo el 16,8% presentaba incapacidad moderada a grave, un porcentaje inferior al identificado en el presente estudio. En el estudio chino, los grados más bajos de incapacidad funcional se asociaron con el deterioro cognitivo, la presencia de síntomas depresivos y los antecedentes previos de ACV, dato que, aunque no presentó significación estadística en nuestra muestra, puede estar relacionado con peores resultados^(21)^.

La muestra estaba compuesta mayoritariamente por personas mayores y ancianos longevos, que tienen más probabilidades de sufrir un ACV al convivir con enfermedades que afectan al sistema cardiovascular. Se estima que el riesgo de ACV se duplica cada 10 años a partir de los 55 años^(22)^. Nuestros resultados corroboran otro estudio^(21)^, que muestra que el 77,1% sufrió un ACVi entre los 61 y los 80 años. A pesar de la independencia entre la edad y la discapacidad, cabe destacar la importancia de la prevención y el control de los factores de riesgo para este evento en todas las etapas de la vida.

El ACVi afectó proporciones similares entre los sexos, con predominio de casos en mujeres, hallazgo que corrobora los datos de un estudio nacional^(15)^. Esta mayor incidencia en el sexo femenino puede estar relacionada con niveles más elevados de lípidos, la menopausia y el impacto del envejecimiento sobre la salud vascular. En el presente estudio, las mujeres presentaron, en mayor proporción, discapacidad moderada a grave, y tienen mayor probabilidad de evolucionar hacia un pronóstico desfavorable, con un riesgo aumentado de muerte^(23)^.

La mayoría de los participantes en el estudio vivían con una pareja. Teniendo en cuenta el elevado porcentaje de personas con discapacidad moderada a grave, se observa una dependencia significativa del apoyo social y la ayuda para el autocuidado. Las personas casadas o en unión estable tienden a recibir más apoyo emocional y práctico de sus cónyuges durante la recuperación tras un ACV isquémico, lo que puede favorecer el cumplimiento de las recomendaciones médicas y la adopción de un estilo de vida saludable, contribuyendo a un pronóstico más favorable^(16)^. Sin embargo, en el presente estudio, las variables estado civil y grado de discapacidad resultaron independientes.

Cabe destacar que el enfermero puede identificar a los cónyuges de las víctimas de ACVi que pueden ofrecer apoyo y participar activamente en la planificación, supervisión y ejecución de los cuidados en el hogar, así como en la obtención de recursos, la motivación y el seguimiento del proceso de rehabilitación. En situaciones de desigualdad social, como era el caso de la mayoría de la muestra, el reto de los cuidados tras un ACVi es aún mayor. La muestra estaba formada predominantemente por personas negras y pardas, con bajo nivel educativo, bajos ingresos y sin actividad laboral. El bajo nivel educativo puede dar lugar a bajos ingresos, menor conocimiento de los factores de riesgo y, en consecuencia, menor prevención y control de la enfermedad^(17)^. Las personas blancas, en general, tienen mayor acceso a la rehabilitación tras un ACVi en comparación con las personas negras y pardas^(17)^. En nuestro estudio, a pesar de la ausencia de significación estadística entre los ingresos y la discapacidad, se identificó un mayor porcentaje de participantes con menor poder económico con discapacidad grave. Por lo tanto, es necesario garantizar, en el proceso de rehabilitación, el acceso continuo a los servicios públicos para los grupos en situación de insuficiencia socioeconómica, con el fin de asegurar la eficacia de su recuperación y la inclusión social.

Los participantes sin actividad laboral presentaron una discapacidad moderada a grave en mayor proporción. Un estudio realizado con personas de Japón mostró un mayor riesgo de morbimortalidad por ACV entre aquellos que pasaron al menos un período sin ejercer actividad laboral o perdieron sus empleos en comparación con los que estaban activos en el trabajo^(24)^. Se puede plantear la hipótesis de que la situación laboral activa propicia el estímulo físico y cognitivo con la recuperación de habilidades y la mejora del estado emocional.

En cuanto a las variables clínicas, un alto porcentaje de participantes tenía hipertensión arterial, un factor de riesgo independiente para el ACV y su recurrencia. Estos presentaron en mayor proporción incapacidad moderada a grave, con una diferencia estadística límite en el análisis bivariado. La hipertensión arterial se asocia a un peor pronóstico funcional, ya que aumenta el riesgo de edema cerebral y hemorragia intracerebral. Las personas hipertensas afectadas por un ACVi tienden a presentar una mayor gravedad del déficit neurológico inicial, evaluado mediante la escala NIHSS, además de una mayor extensión de la lesión cerebral y tasas más elevadas de incapacidad funcional en comparación con las personas normotensas^(25)^.

Una menor proporción de participantes presentó fibrilación auricular, mientras que la diabetes mellitus afectó a aproximadamente una cuarta parte de la muestra y la dislipidemia, a aproximadamente un tercio. Estas afecciones clínicas se asocian con un mayor riesgo de morbimortalidad por ACVi, ya que potencian el daño micro y macrovascular^(26)^ y requieren un manejo clínico continuo. Sin embargo, en el presente estudio, estas variables se mostraron independientes del grado de incapacidad funcional. Cabe destacar la importancia del diagnóstico preciso de la fibrilación auricular después del ACVi, ya que permite implementar estrategias específicas de prevención secundaria, como el uso de anticoagulantes, que contribuyen a reducir el riesgo de recurrencia^(27)^.

Durante el periodo de seguimiento, algunos participantes fueron diagnosticados con COVID-19; sin embargo, no se observó ninguna asociación entre la infección y el grado de incapacidad funcional. Aun así, la literatura señala que los individuos que contraen COVID-19 después de un ACV presentan una mayor propensión a la recurrencia del evento, formas más graves de la enfermedad y complicaciones cardiovasculares^(28)^.

Aproximadamente un tercio de la muestra presentaba antecedentes de ACV, lo que se asoció con un mayor grado de discapacidad solo en el análisis bivariado. Por otro lado, la recurrencia del ACVi durante el periodo de seguimiento fue una de las tres variables significativamente asociadas con la discapacidad moderada a grave en el análisis multivariado, aumentando en 3,82 veces la probabilidad de disfunción funcional. Corroborando estos hallazgos, Yang y colaboradores^(29)^ demostraron, mediante regresión logística múltiple, que la recurrencia del ACV es predictiva de un mayor grado de discapacidad. El ACVi es responsable de discapacidades agudas que tienden a estabilizarse después de un período de tres a seis meses. Sin embargo, la estabilización se ve afectada si se producen nuevos eventos.

Los antecedentes de ACV y su recurrencia, observados en nuestro estudio, pueden conducir al agravamiento de los déficits relacionados con la marcha y al deterioro de la función motora debido a nuevas lesiones cerebrales^(29)^. La recurrencia del ACVi en un plazo de dos a tres años después del accidente cerebrovascular puso de manifiesto las dificultades en la prevención y el control de la enfermedad y la importancia del seguimiento continuo de las personas afectadas por parte de un equipo multidisciplinar, la evaluación y el apoyo para la adherencia al tratamiento. Una cohorte prospectiva con 764 pacientes observó que el 18,0% sufrió un nuevo ACVi en los tres primeros años tras el alta, lo que corrobora nuestros resultados, siendo la falta de adherencia a la profilaxis secundaria tras el primer evento un factor de riesgo de recurrencia^(12)^.

En la presente investigación, el déficit neurológico moderado evaluado por la NIHSS fue más frecuente. En la escala, a medida que aumenta la puntuación, se expresa un mayor compromiso neurológico y discapacidad, lo que se traduce en resultados desfavorables para la calidad de vida^(16)^. En el análisis bivariado, la discapacidad moderada a grave aumentó proporcionalmente en función de la gravedad del evento. El análisis multivariado hizo más evidente la relación establecida entre la gravedad del déficit neurológico y el grado de discapacidad, ya que las personas con déficit neurológico moderado y grave tuvieron, respectivamente, 2,35 y 4,40 veces más discapacidad moderada a grave en comparación con aquellas con discapacidad leve. Una cohorte de cinco años evaluó a personas con y sin discapacidad funcional después de un ACVi, y mostró que el 45% de la muestra (n = 893) seguía teniendo discapacidad funcional después de ese período y que el NIHSS más alto se asociaba significativamente con un mayor grado de discapacidad, lo que corrobora nuestros hallazgos^(29)^. Otro estudio, realizado con 101 personas que sufrieron un ACVi, evaluadas a uno, tres y seis meses del evento, observó que, seis meses después del ACV, el 50% no logró recuperar sus funciones y tenía puntuaciones más altas en el NIHSS^(16)^.

La mayoría de los participantes llegaron al lugar del estudio dentro de la ventana terapéutica; sin embargo, menos del 30% se sometió a trombólisis, lo que contribuyó a un mayor grado de discapacidad, como mostró el análisis multivariante. Los participantes que no se sometieron a trombólisis tuvieron 3,0 veces más discapacidad moderada a grave en comparación con los que recibieron el tratamiento trombolítico, lo que evidencia sus beneficios a los dos y tres años del evento. Además, un porcentaje significativo llegó fuera de la ventana terapéutica, dejando de disfrutar de sus beneficios. Actualmente, las directrices para el ACV limitan el inicio de la terapia trombolítica intravenosa a 4,5 horas después del inicio de los síntomas o del *wake up stroke*. El uso de la terapia con alteplasa muestra que las personas beneficiadas presentaron déficits neurológicos mínimos o ausentes^(1)^. Otros autores reforzaron los efectos positivos de la trombólisis, ayudando a reducir la incapacidad, aumentando la probabilidad de un retorno más rápido al trabajo y promoviendo la mejora de la calidad de vida^(30)^.

Se refuerza la importancia de la actuación del enfermero en el apoyo al paciente y a la familia desde el inicio del proceso de rehabilitación, especialmente en la fase de transición del hospital al domicilio. En este contexto, corresponde al profesional identificar a los cuidadores potenciales y capacitarlos para ofrecer un apoyo adecuado al paciente. Este proceso requiere adaptación tanto por parte del paciente como de la familia, sobre todo en los casos de mayor grado de incapacidad. Las orientaciones compartidas por el enfermero aún en el entorno hospitalario son fundamentales no solo para el cuidado domiciliario, sino también para garantizar la continuidad del seguimiento en la atención básica, promoviendo el vínculo con la unidad de salud^(31)^.

Los resultados del presente estudio indicaron que un mayor déficit neurológico, la ausencia de trombólisis y la recurrencia del ACVi fueron los principales factores asociados a la incapacidad moderada a grave. Estos hallazgos resaltan la importancia de las políticas públicas dirigidas al cuidado de las personas afectadas por el ACVi, así como de estrategias efectivas de rehabilitación en todas las fases del tratamiento, con el objetivo de minimizar el grado de incapacidad funcional. Se refuerza la necesidad de estudios orientados al seguimiento longitudinal de estos individuos por equipos multiprofesionales en el sistema público de salud, garantizando el acceso universal a una rehabilitación de calidad y a medidas eficaces de prevención secundaria.

Los resultados refuerzan la importancia de adherirse a estrategias consolidadas de prevención, como la iniciativa HEARTS, que representa un eslabón fundamental en la prevención de las enfermedades cerebrovasculares. Se trata de un acrónimo que engloba seis pilares esenciales: hábitos saludables; protocolos clínicos basados en la evidencia; acceso a medicamentos, tecnologías e insumos; manejo del riesgo cardiovascular; actuación en equipo multidisciplinario; y monitorización mediante indicadores estandarizados. La implementación efectiva de esta estrategia puede contribuir significativamente a mejorar los resultados en la prevención y el control del ACVi.

Los resultados de esta investigación también evidencian la necesidad de ampliar la cobertura del sistema público de salud en lo que se refiere al uso de la terapia trombolítica, eficaz en la reducción de las disfunciones neurológicas más graves. Paralelamente, es fundamental que la comunidad y las personas afectadas reciban orientación para reconocer precozmente los signos y síntomas del ACV isquémico, con el fin de buscar atención en unidades de referencia en neurología dentro de la ventana terapéutica. En el presente estudio, aproximadamente el 40% de los participantes llegaron al servicio hospitalario fuera de la ventana terapéutica. Aquí reside un espacio fundamental para la actuación del enfermero a través de actividades de educación en salud. Además, un país con menos disparidades sociales sería capaz de garantizar mejores condiciones de vida, minimizando la morbilidad y el sufrimiento resultante.

Este estudio presenta algunas limitaciones que deben tenerse en cuenta al interpretar los resultados. Se produjo una pérdida de participantes durante el seguimiento, lo que puede comprometer la representatividad de la muestra final. Además, la recolección de datos mediante entrevistas telefónicas y, en algunos casos, en los que los datos fueron proporcionados por cuidadores y familiares, puede haber generado algún sesgo en la información. Otro sesgo a tener en cuenta se refiere a la selección de los participantes en la línea de base, ya que se excluyó a algunos pacientes en condiciones más graves o con déficits cognitivos que no contaban con acompañantes disponibles en el momento de la inclusión, lo que puede limitar la generalización de los hallazgos a la población con mayor compromiso funcional.

## CONCLUSIÓN

El estudio mostró el impacto del ACVi en la vida de las personas afectadas, con más de un tercio con discapacidad moderada a grave entre dos y tres años después del ACV. Las variables asociadas a la discapacidad moderada a grave, en el análisis multivariante, fueron la mayor gravedad del déficit neurológico, la no realización de trombólisis y la recurrencia del AVCi. El estudio muestra la necesidad de avances en la red de atención sanitaria, garantizando la ampliación de la oferta de trombólisis, el acceso a unidades de tratamiento especializadas y la rehabilitación precoz y continuada, con un equipo multidisciplinar. Destaca que son fundamentales las políticas e intervenciones efectivas orientadas a la promoción de la salud, la prevención y el control de los factores de riesgo de ACVi, además del estímulo a la presentación precoz de las víctimas en un hospital de referencia en neurología para minimizar las repercusiones negativas del ACVi en la vida personal, familiar y para la sociedad en general.

## Data Availability

El conjunto de datos que respalda los resultados de este estudio no está disponible públicamente.
